# Novel Low-y⁺ Roughness Correlation for Hydrogen-Fired Jet Burners Validated Under Isothermal and Reacting Conditions

**DOI:** 10.1007/s10494-026-00799-z

**Published:** 2026-10-06

**Authors:** Robin Vivoli, Daniel Pugh, Anthony Giles, Burak Goktepe, Tomas Davies, Steven Morris

**Affiliations:** School of Engineering, College of Physical Sciences and Engineering, Cardiff University, Queen’s Building, Cardiff, CF24 3AA UK

**Keywords:** Surface roughness, Additive manufacturing, Hydrogen combustion, Detached Eddy Simulation, Gas turbine

## Abstract

Surface roughness has a critical influence on the aerothermal and combustion behavior of gas turbine burners, particularly with the growing use of additively manufactured (AM) components that exhibit inherently rougher surfaces than conventionally machined parts. This study investigates the impact of surface roughness on flow and flame dynamics in a premixed jet burner (PJB) operating under both isothermal-air and reacting hydrogen-fired conditions. Experimental measurements using Laser Doppler Anemometry (LDA) and OH* chemiluminescence were complemented by Computational Fluid Dynamics (CFD) simulations employing Reynolds-Averaged Navier–Stokes (RANS) and Detached Eddy Simulation (DES) frameworks. Building upon existing equivalent sand-grain roughness (kₛ) models, a novel correlation incorporating both measured roughness parameters and bulk flow velocity was developed to improve predictive accuracy for resolved boundary layers (y^+^ ≈ 1), a regime in which k_s_^+^ > y⁺ is common and standard roughness-function formulations lose validity. The new correlation demonstrated strong agreement with experimental data in isothermal simulations. When extended to reacting hydrogen–air flames, RANS simulations successfully reproduced roughness-induced flow redistribution and flame shortening, although DES tended to overpredict bluff-body effects and underperform under lean conditions. Overall, the proposed k_s_ correlation, validated under isothermal conditions, provides a computationally efficient framework for modelling surface-roughness effects, and its extension to reacting hydrogen flames, particularly under RANS, offers a promising basis for future development toward additively manufactured gas turbine components.

**Supplementary Information:**

The online version contains supplementary material available at https://doi.org/10.1007/s10494-026-00799-z.

## Introduction

Surface-roughness-induced effects have been shown to significantly influence the performance and operability of both legacy swirl burners (Psomoglou et al. 2024; Runyon et al. 2020) and novel jet burner architectures (Vivoli 2025; Vivoli et al. 2026). These effects having been reported for CH₄ (Runyon et al. 2020) and CH₄/H₂ (Psomoglou et al. 2024) fuel blends in swirl burners, and for H₂ in jet-burner configurations (Vivoli 2025; Vivoli et al. 2026). As additive manufacturing (AM) becomes increasingly adopted for gas turbine components such as burners and subassemblies (Andersson et al. 2017; Durocher et al. 2024; Navrotsky et al. 2015), the inherently poorer surface finish of as-built AM parts compared to conventionally manufactured ones (Calignano et al. 2013; Shinonaga et al. 2023) underscores the need for robust and cost-effective numerical tools to assess roughness effects.

This is particularly relevant given increased surface roughness has been demonstrated to enhance flashback resistance in both swirl-stabilized (Al-Fahham et al. 2017) and premixed jet burner (Vivoli et al. 2026) architectures, with the latter attributed to roughness-induced turbulence and boundary-layer velocity modifications (Vivoli 2025; Vivoli et al. 2026). Furthermore, recent 2D and 3D direct numerical simulations (Ding et al. 2021; Zhu et al. 2026) together with experimental work (Jaeschke et al. 2026) have shown surface roughness to intensify wall heat losses in combustion settings.

With hydrogen gaining prominence as a zero-carbon energy carrier (McCay and Shafiee 2020) and substantial R&D efforts directed toward developing lean-premixed combustion systems capable of operating with up to 100% hydrogen (Cecere et al. 2023; Kim 2019; Toit et al. 2018), addressing challenges associated with its high reactivity and consequent flashback propensity (Ebi et al. 2021; Kıymaz et al. 2022) has become a key research priority. Understanding how surface roughness can help mitigate such risks is thus of considerable industrial interest.

Computational Fluid Dynamics (CFD) provides a powerful framework for investigating roughness effects by enabling detailed flow and flame analysis in regions difficult to probe experimentally. However, accurately reproducing these effects numerically remains challenging (Bons 2010; Stripf et al. 2008; Vivoli et al. 2025b). While high-fidelity techniques such as discrete-element models (DEM) (Aupoix 2016; McClain et al. 2004) or fully resolved roughness representations (Bons et al. 2008; Kapsis et al. 2020) can capture surface effects with precision, their computational expense limits practical applicability. Consequently, empirical approaches that correlate measured surface metrics (e.g. Ra, Rq, Rz) to an “equivalent sand-grain” roughness (*k*_*s*_) (Krasilnikov et al. 2023; Lu and Liou 2007) remain widely used. Yet, this method depends heavily on the suitability of the chosen *kₛ* correlation, which often requires case-specific calibration and cannot generally be expected to yield physically representative predictions of both flow field and wall heat transfer simultaneously (Bons 2010). Moreover, limited studies have explored the use of such correlations under reacting conditions (Vivoli et al. 2025b).

### Research Scope

The present study aims to model surface-roughness effects using a *kₛ* correlation in conjunction with a high physical-resolution approach featuring a resolved boundary layer (y^+^ ≈ 1). The performance of existing *kₛ* correlations being assessed before developing a novel, case-specific correlation. The correlation is derived and fully validated against flow-field measurements under isothermal-air conditions, and subsequently applied without modification to reacting hydrogen–air conditions to assess whether it remains broadly representative of roughness-induced effects on flow and flame behavior. For the purpose of validation, both reacting H_2_ and isothermal-air datasets are collated. The former were acquired at thermal powers of 15, 20, and 25 kW for equivalence ratios (φ) of 0.5, 0.7, and 0.9. These conditions were chosen based on prior commissioning work (Vivoli et al. 2026) which confirmed stable burner operation within this range. Corresponding isothermal-air datasets were obtained under equivalent total air mass flow conditions, with bulk velocities ranging from 20 to 64 m/s (24,000 < Re < 80,000), providing a broad validation matrix for assessing both existing and newly derived roughness correlations.

## Materials and Methods

In this work, both experimental facilities and diagnostics, as well as numerical methodologies, are employed. The following subsections present these two aspects separately: one detailing the experimental setup and procedures, and the other describing the numerical methods.

### Experimental and Diagnostic Setup

#### Premixed Jet Burner

**Fig. 1:**
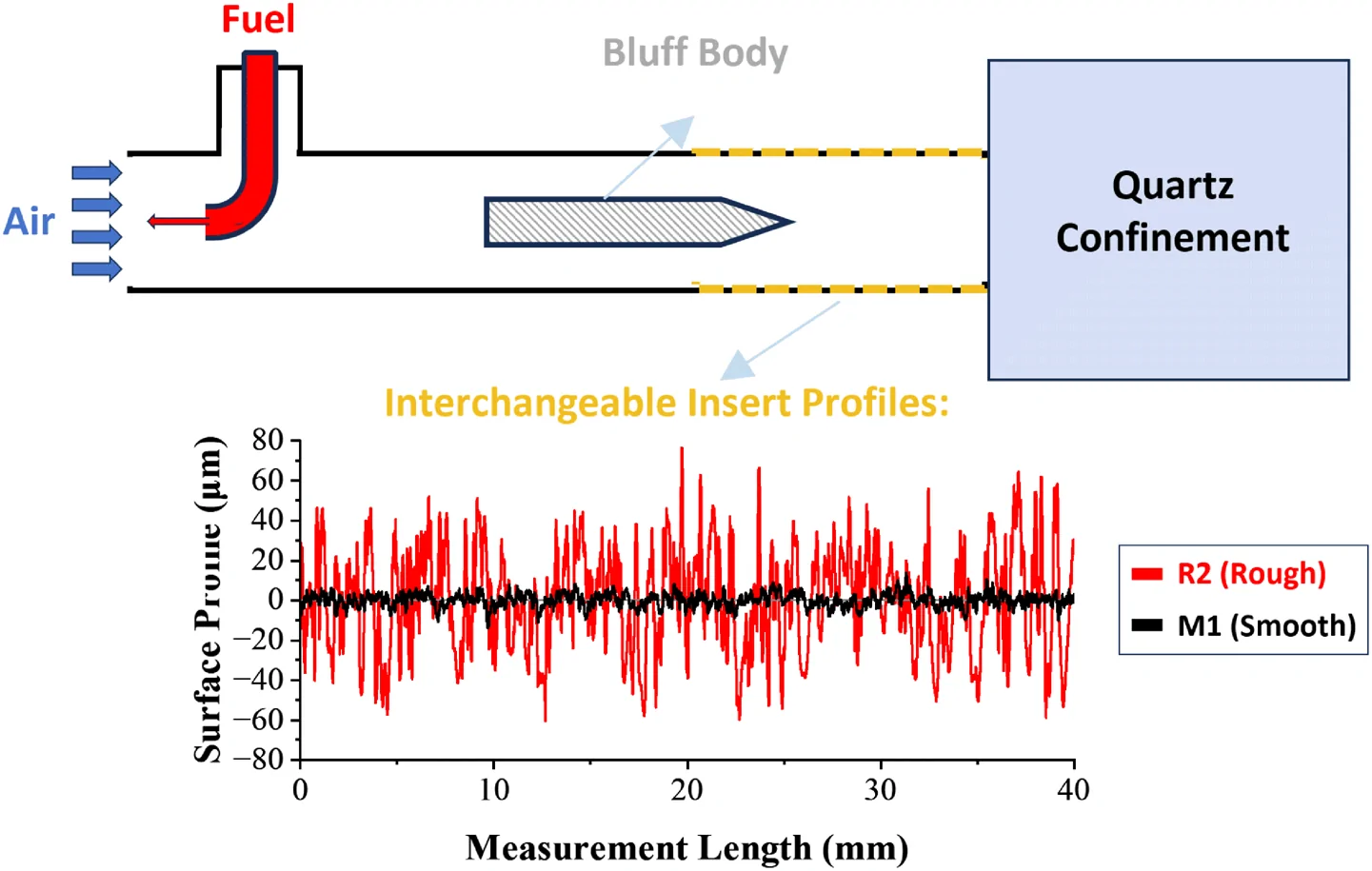
PJB schematic with detail showing the surface profiles of the two roughness inserts tested

A newly commissioned premixed jet burner (PJB), a schematic of which is shown in Fig. 1, was employed in this study. This burner was inspired by industrial combustor architectures (Krebs et al. 2022) and employed a fully premixed configuration to eliminate fuel-air mixing variability, thereby simplifying both the experimental rig and the numerical setup. The burner incorporates interchangeable inserts forming the final 200 mm of the premixing channel, enabling isolated assessment of surface-roughness effects.

Two interchangeable inserts, the surface profiles of which are shown in Fig. 1, were tested in this work: ‘*M1*’, conventionally manufactured, and ‘*R2*’, EDM-textured to replicate angled downskin surfaces typical of “worst-case” AM conditions (Koutiri et al. 2018; Subramanian et al. 2021). Flow testing (Vivoli et al. 2026) showed bulk velocity variations between inserts below 2%, indicating any performance differences stem from surface roughness effects rather than geometric discrepancies. Further details regarding burner configuration, insert design, manufacturing, and surface topology assessment are provided elsewhere (Vivoli et al. 2026). Average roughness parameters are summarized in Table 1 for clarity. Ra (arithmetic mean roughness) reflects overall texture magnitude, Rq (root mean square roughness) is more sensitive to large deviations due to squaring, and Rz (ten-point average roughness) captures mean peak-to-valley height, making it more representative of extreme surface features (British and Standard 2010).

**Table 1:** Surface roughness measurements for each insert

	*Ra* (µm)	*Rz* (µm)	*Rq* (µm)
M1	1.73	9.33	2.13
R2	21.2	132.33	26.32

From Table 1, both M1 and R2 exhibit an Rq/Ra ratio of approximately 1.23–1.24, which is close to the theoretical value of 1.25 expected for a Gaussian height distribution indicating statistically random roughness (Thomas 1998). Notably, although R2 shows roughness values approximately an order of magnitude higher than M1, the similar Rz/Ra and Rq/Ra ratios indicate a comparable roughness distribution shape, with R2 representing an amplified but qualitatively similar surface texture.

#### Flame Characterization

Changes in flame structure were characterized via high-speed OH* chemiluminescence (CL) focusing on the well-known A²Σ⁺ → X²Π OH* system (Gaydon 1974). CL data were acquired at 4 kHz via a 315 nm (± 15 nm FWHM) narrow bandpass filter, with further information on the high-speed imaging setup presented in other works (Pugh et al. 2021, 2024; Runyon et al. 2020). Each dataset was temporally averaged over 2000 instantaneous images and flame centroids were extracted to identify roughness-induced shifts, following the methodology established in previous studies (Runyon 2017; Vivoli et al. 2026).

#### Isothermal Flow Characterization

**Fig. 2:**
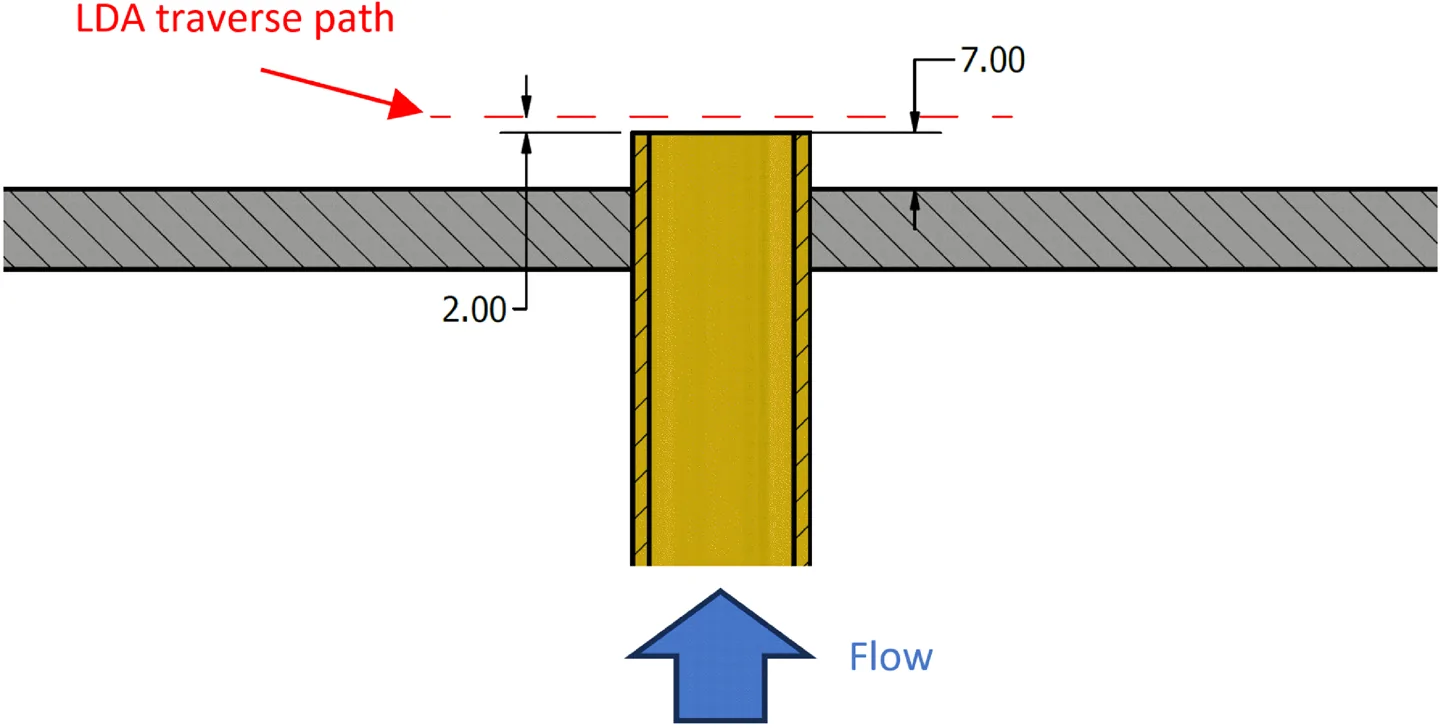
Section view of the unconfined rig with LDA traverse path shown in red. All dimensions in mm

A 1D Dantec Dynamics Flowlite Laser Doppler Anemometry (LDA) System, detailed in previous studies (Runyon et al. 2020; Vivoli et al. 2026), was used for characterizing the mean flow field and turbulence characteristics of isothermal airflow exiting the burner nozzle. In this study both mean (u) and fluctuating (u′_RMS_) axial velocities were measured 2 mm downstream of the nozzle exit with the quartz confinement removed as shown in Fig. 2.

### Numerical Setup

As stated at the outset, this work aims to model surface-roughness effects on a resolved boundary layer (y^+^ ≈ 1). It is important to note that the objective of this work is not to develop a roughness correlation specific to low-y⁺ meshes per se. Rather, the proposed *k*_*s*_ correlation was developed for wall-resolved simulations in which the resulting $\:{k}_{s}^{+}$ may exceed the first-cell y⁺ ($\:{k}_{s}^{+}$ > y^+^), a regime where many commercial CFD solvers are unable to capture physically meaningful roughness effects (Eça and Hoekstra 2011; Suga et al. 2006).

Although Large Eddy Simulation (LES) is widely adopted in turbomachinery research, the employed roughness-modelling framework is not readily compatible with LES formulations (Siemens Digital Industries Software 2021). Detached Eddy Simulation (DES), however, provides a suitable compromise by applying LES in core flow regions while retaining RANS and its associated roughness treatments near walls (Pan and Zhang 2023; Vivoli et al. 2025b). Accordingly, both steady-state (RANS) and unsteady (DES) simulations were employed to capture surface-roughness effects. RANS simulations, with their lower computational cost, were used during the development phase, while DES was applied to a limited set of cases to evaluate whether the novel correlation remains valid under higher physical resolution conditions.

**Fig. 3:**
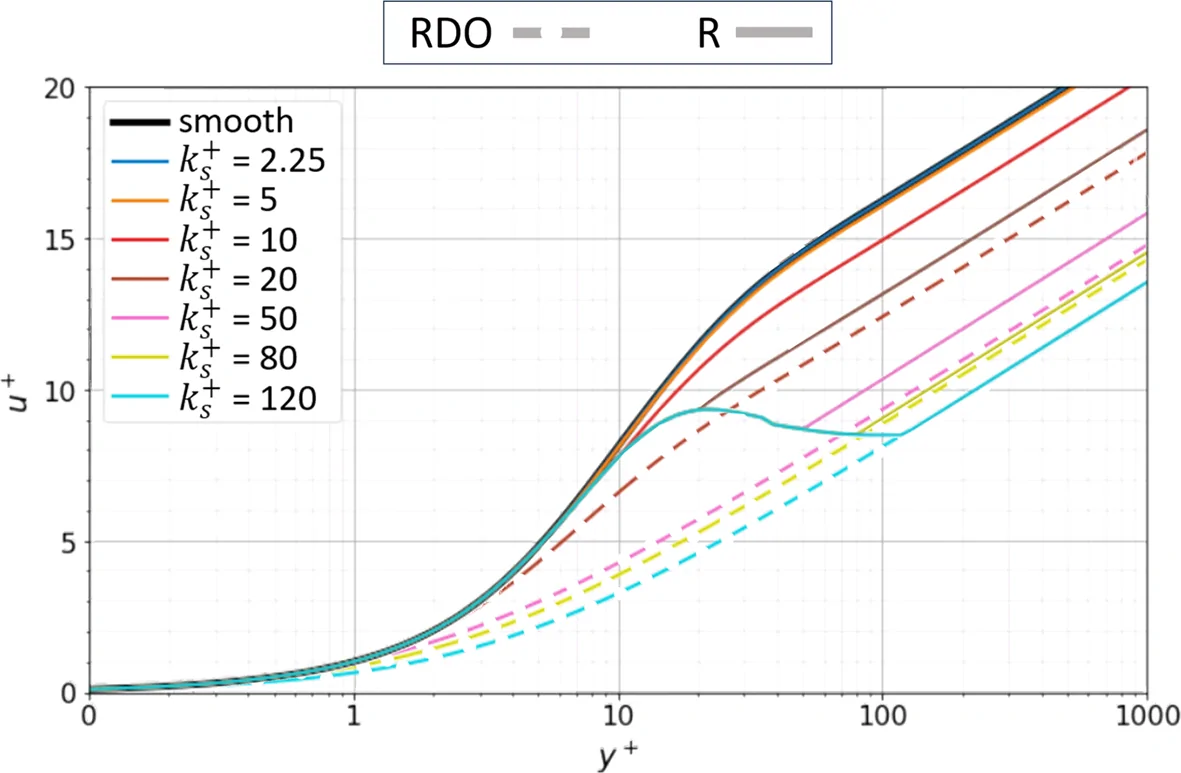
Changes in u+ velocity profiles for different $\:{k}_{s}^{+}\:$ values when utilizing the R and RDO approaches. (modified from Simcenter STAR CCM+(Simcenter STAR CCM+ 2025))

From previous swirl burner studies (Vivoli et al. 2025b), the k-ε Elliptic Blending (EB) model was selected for its superior treatment of wall roughness with a resolved boundary layer. Unlike the standard roughness model (R) (Jayatilleke 1969), which shifts the log-law via a roughness function and is therefore only strictly valid once the near-wall cell lies outside the roughness sublayer (y^+^ ≳ $\:{k}_{s}^{+}$), EB uses the Rough Displaced Origin (RDO) approach, computing a $\:{k}_{s}^{+}$-dependent displaced origin $\:{y}_{0}^{+}$ at the boundary condition level (Durbin et al. 2001; Knopp et al. 2009). As shown in Fig. 3, the RDO approach therefore allows for meaningful roughness effects to be captured even for *y*^*+*^ < $\:{k}_{s}^{+}$ albeit at the cost of reduced roughness sensitivity for $\:{k}_{s}^{+}$ < 20 (Simcenter STAR CCM+ 2025).

We note that a fully consistent treatment - extending this displaced-origin idea to the off-wall boundary conditions of the turbulence model, as demonstrated by Prakash and Laurendeau (2020) - would remove this residual y⁺ dependence entirely; however, that requires specialist solver-level modification of the turbulence-model equations, access to which is not typically provided in commercial CFD frameworks. We therefore rely on the vendor-implemented RDO formulation used here as the closest available approximation. Accordingly, EB was employed in this study for both RANS and DES (DDES) simulations, using STAR-CCM+ v23.02.

The Reynolds analogy, the assumption that momentum and heat transfer are governed by the same near-wall transport mechanism, is known not to hold for rough surfaces since heat transfer remains conduction-limited while momentum transfer is dominated by form drag (Cope 1941; Dipprey and Sabersky 1963; Owen and Thomson 1963). As a result, several thermal correction approaches have been developed to address this (Aupoix 2015; Chedevergne 2018; Prakash and Laurendeau 2020). In the present solver, the EB RDO thermal wall treatment applies the same $\:{y}_{0}^{+}$ parameter used for the momentum equation directly to the temperature profile, without an independent roughness-dependent correction - a simplification consistent with the Reynolds analogy. Consistent with the limitation of a single *k*_*s*_ value noted in Sect.  1, having obtained physically representative flow field effects, it is likely that thermal effects will be overestimated (Bons 2005; Dipprey and Sabersky 1963). As a result, two separately calibrated momentum and thermal roughness correlations are required. In STAR-CCM+, however, for a fluid-only domain, as utilized in this work, a single *k*_*s*_ value must serve both the momentum and thermal wall treatments simultaneously. As stated at the outset, the present work develops and validates a single *k*_*s*_ correlation targeted at flow field behavior under isothermal conditions; it is applied unmodified under reacting conditions to assess whether roughness effects on the flow field are still broadly captured, and no claim is made regarding quantitative accuracy of the associated heat transfer predictions. A fully independent validation of roughness-induced heat transfer would require a dedicated, independently-calibrated thermal roughness correlation, or a solver permitting turbulence-model-level modifications such as those demonstrated by Prakash and Laurendeau (2020).

Whilst key differences exist in the isothermal and reacting flow domain and underlying physics, details of which are provided in subsections 2.2.1 and 2.2.2 respectively, consistent methodologies were adopted for mesh generation. For the RANS cases, mesh independence studies were performed as outlined in previous work (Vivoli et al. 2025b) (results compiled in the supplementary data). Converged results were obtained for meshes containing ~ 2 × 10⁶ elements for isothermal cases and ~ 2.7 × 10⁶ elements for reacting cases, with only minor boundary-layer adjustments made to maintain *y⁺* ≈ 1 across all conditions. Converged RANS data were then used to guide mesh refinement for the DES cases, following approaches in the literature (Vivoli et al. 2025a, b). Depending on the flow rate and physical conditions, DES mesh sizes ranged from approximately 8.8 × 10⁶ to 9.5 × 10⁶ elements.

#### Isothermal Domain and Physics

**Fig. 4:**
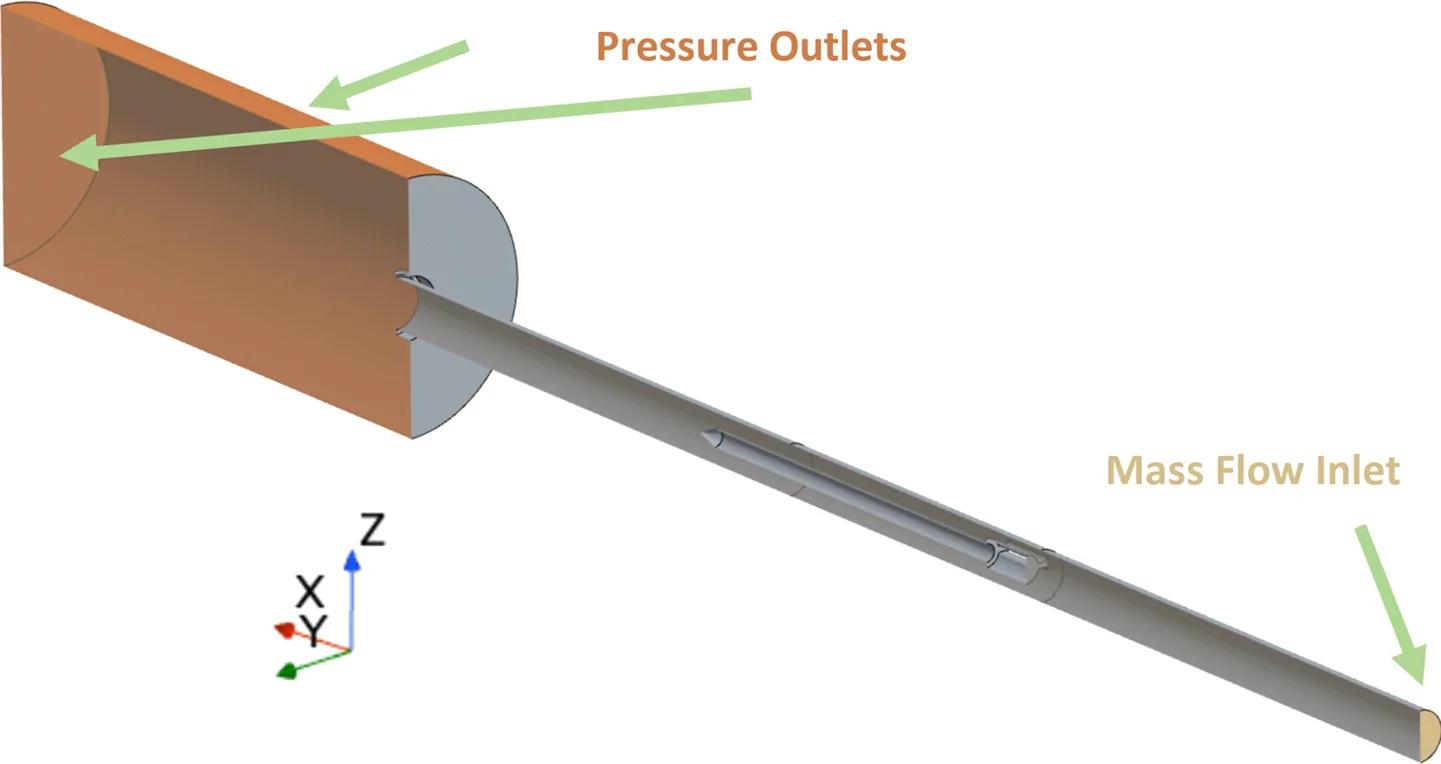
Isothermal computational domain with 5x expansion ratio. Inlet and outlet surfaces highlighted

To capture the unconfined nature of the isothermal burner-exit flow while limiting computational cost, exploratory simulations (summarized in the supplementary material) indicated that an expansion ratio of five times the nozzle inner diameter provides an optimal compromise. Because the fuel line was inactive during isothermal tests, it was omitted to simplify meshing and reduce cell count; the premixing chamber was instead extended 200 mm upstream of the bluff body to ensure adequate flow development. A mass flow inlet was applied to the base of the premixing section with pressure outlets applied to the top and perimeter of the combustion chamber as shown in Fig. 4.

The isothermal physics was set up as follows; the Segregated Flow Isothermal model was utilized and the reference pressure set to atmospheric (0.101 MPa). The default STAR CCM+ material properties for air were left unchanged. Inlet conditions together with naming convention utilized for all isothermal cases are collected in Table 2.

**Table 2:** Inlet and outlet conditions for numerical isothermal air simulations

Naming Convention	Airflow (g/s)	Reacting Equivalent Condition	ū (m/s)	Ambient Temperature (K)
Iso_20	6.1	15 kW @ φ = 0.93	19.5	286
Iso_32	10	20 kW @ φ = 0.7	32.1	
Iso_40	12.65	25 kW @ φ = 0.7	40.5	
Iso_64	19.97	25 kW @ φ = 0.4	64	

Time-step and flow through time (FTT) choices for the DES simulations were informed utilizing the respective RANS simulations as described in other works (Vivoli et al. 2025a, b). Simulations ran for 1 FTT before initiating time-averaging which was performed for a maximum of 5 FTTs. Owing to the high computational cost of DES and the requirement for increased mesh resolution at lower flow speeds, where turbulence intensity increases with decreasing Reynolds number (Varadharajan 2017), the effectiveness of roughness correlations was only assessed on the highest bulk flow velocity case with the temporal parameters collected in Table 3.

**Table 3:** Temporal settings for isothermal DES simulation

	Iso_64
FTT (s)	0.023
Total Time (s)	0.14
Inner Iterations	20
Time-Step (µs)	6

#### Reacting Domain and Physics

**Fig. 5:**
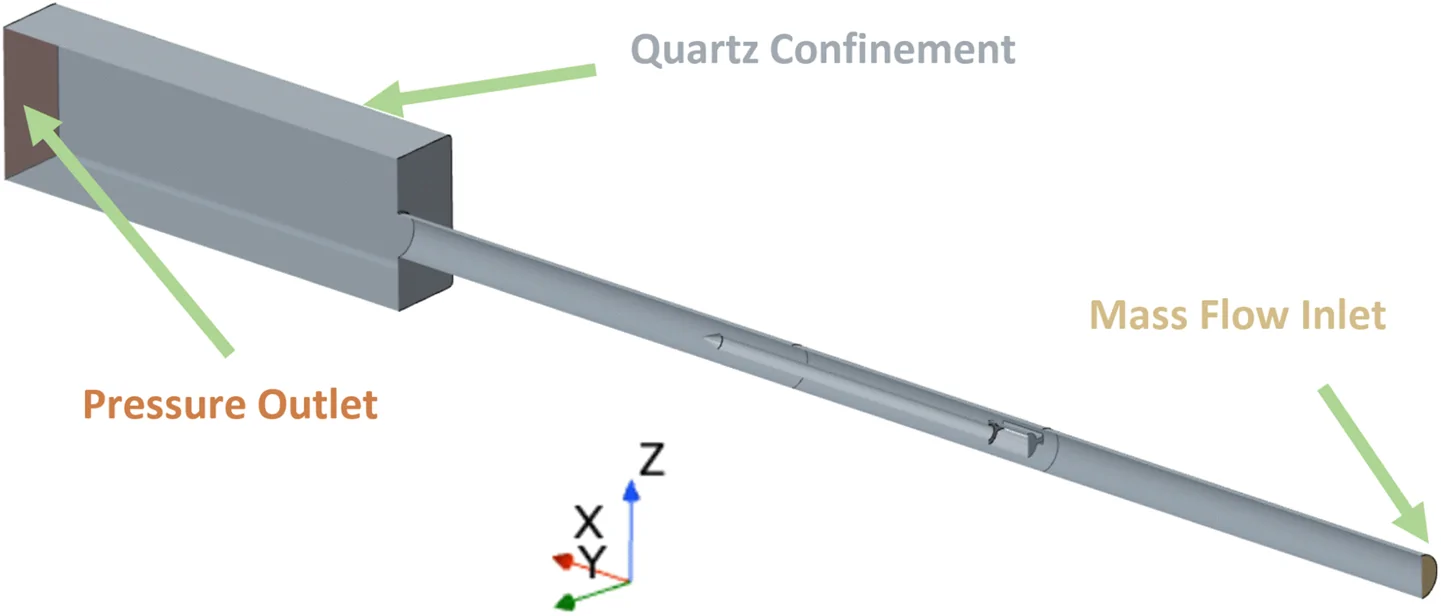
Reacting PJB numerical domain

For the reacting domain the square quartz confinement was retained. Preliminary RANS results, available in the supplementary data, showed adequate premixing upstream of the chamber, allowing fuel injection to be omitted. As shown in Fig. 5, a mass flow inlet was thus positioned at the base of the model 200 mm upstream of the bluff body assembly, while a pressure outlet was placed at the end of the quartz confinement to replicate the experimental setup.

The primary objective of this study is not exact replication of absolute experimental flame characteristics, but rather assessment of the relative changes induced by surface roughness under otherwise identical modelling conditions. As such, compromises were made between chemical and physical modelling fidelity and computational cost, given the large number of simulations required for this study. Consistent with this approach, the Ó Conaire (Conaire et al. 2004) mechanism with incorporated OH^*^ (Burcat 2006; Kathrotia 2011) was used, consistent with other works (Panoutsos et al. 2009; Vivoli et al. 2025a), and chosen for its reduced species set. Complex Chemistry, with segregated flow enthalpy, was used together with the Eddy Dissipation Concept (EDC) for turbulence–chemistry interaction. Species diffusion was calculated using the mixture-averaged (Fick’s Law) approximation, the default solver treatment, in order to reduce computational cost. The Soret effect was not enabled in the present simulations, consistent with prior work suggesting that, while thermal diffusion can have a non-trivial local impact, it is not expected to be a leading-order effect for hydrogen flames of this type, nor to alter the overall conclusions of such studies (Howarth et al. 2023). A fixed turbulent Schmidt number of 0.7 was adopted based on Feng et al. (2024), who found this value to give the closest overall agreement with LES results across the domain for micro-tube and free-jet hydrogen/air mixing configurations geometrically similar to those studied here. The EDC fine-structure length-scale factor was increased to 5.4, calibrated against the smooth-wall baseline case to improve agreement with experimentally measured flame characteristics, and held constant across all subsequent smooth- and rough-wall simulations. As the modelling simplifications discussed above were applied identically and held fixed across all smooth- and rough-wall cases, their influence is expected to act as a broadly systematic bias rather than a roughness-dependent one, such that the relative rough-to-smooth comparisons central to this work are expected to be comparatively robust to these simplifications.

Wall temperatures were set in accordance with experimental measurements and, as such, roughness induced changes in heat transfer were not directly modelled due to limitations outlined in Sect.  2.2. All boundary-condition details, including thermocouple measurements, are provided in the Supplementary Data. The reference pressure was set to atmospheric (0.101 MPa).

Time-step and FTT choices for the DES simulations followed the same procedures as Sect.  2.2.1. Simulations ran for 2 FTT before initiating time-averaging which was performed for a maximum of 5 FTTs. Due to the high computational cost associated with DES simulations, the evaluation of roughness correlations was limited to the boundary thermal power conditions of 25 kW. Temporal parameters are collected in Table 4.

**Table 4:** Temporal settings for 25 kW reacting DES simulations

	φ = 0.5	φ = 0.7	φ = 0.9
FTT (s)	0.0176	0.0219	0.0261
Total Time (s)	0.1235	0.1536	0.1824
Inner Iterations	20		
Time-Step (µs)	5.1	6.6	8.0

## Experimental Results

**Fig. 6:**
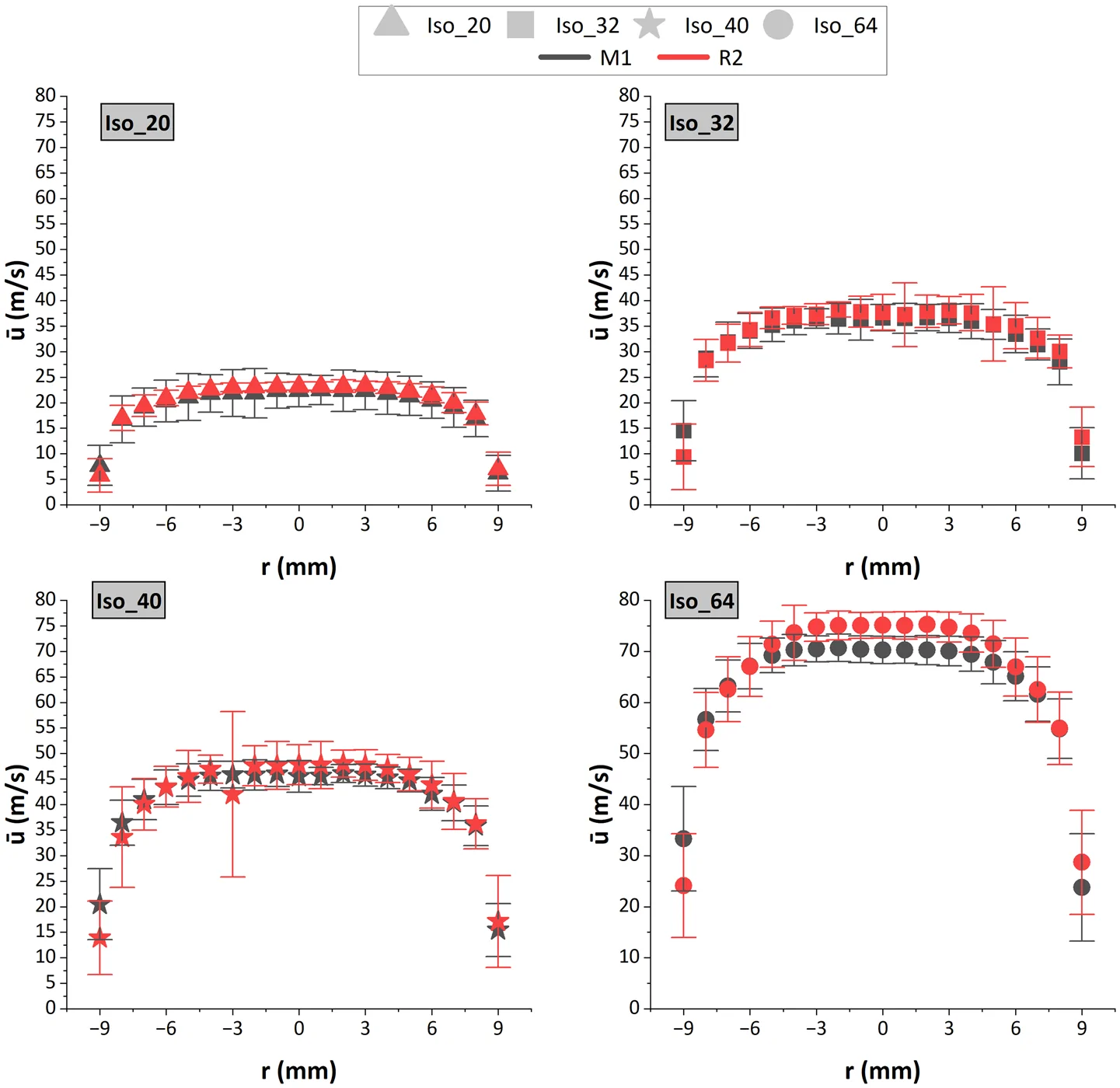
Experimental isothermal axial velocity profiles at various bulk flow velocities. Error bars representing statistical uncertainty via velocity fluctuation measurements (u_RMS_)

Surface roughness has been observed to increase near wall turbulence, reduce near wall velocities, and increase centerline velocities (Schlichting 1979; Vivoli et al. 2026). These effects, previously confirmed by Vivoli et al. (2026), were further expanded on in this experimental campaign as shown in Fig. 6. Given the inlet air temperature was close to ambient (286 K), the wall-to-bulk temperature difference, and hence any roughness-induced changes in wall heat transfer, is expected to be small under these isothermal conditions.

From Fig. 6, it can be observed that, within the inner diameter of the burner exit nozzle (± 9 mm), the rough-wall velocity profiles consistently exhibit lower near-wall velocities and higher centerline velocities compared to corresponding smooth cases. The offset between the smooth and rough centerline velocities increases with bulk flow rate, rising from approximately 4% for the Iso_20 case to about 6.5% for the Iso_64 case. Velocity fluctuations decrease with increasing bulk velocity, which can be partly attributed to the previously discussed increase in turbulence intensity with decreasing Reynolds number (Varadharajan 2017). For the Iso_40 case, an outlier is observed in the R2 velocity profile at a radial coordinate of *r* ≈ − 3 mm. This data point is considered anomalous due to its unusually low velocity (~ 11% lower than adjacent values) and its significantly higher associated statistical uncertainty. It was therefore removed before performing comparisons with numerical datasets.

**Fig. 7:**
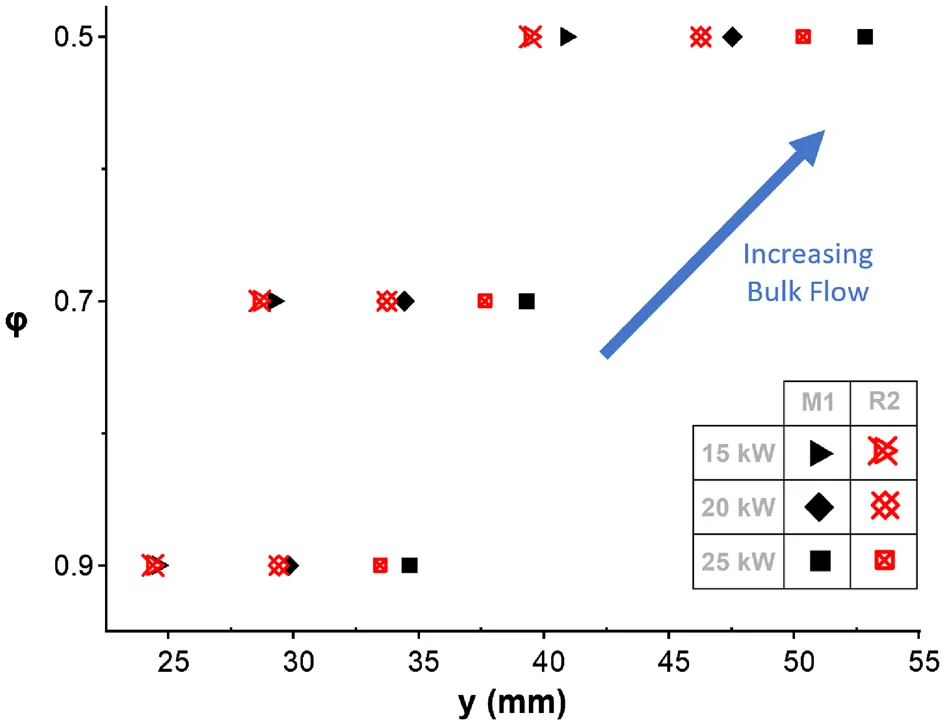
Experimental axial flame centroid locations

From a flame shape standpoint, although both radial (*r*) and axial (*y*) flame centroid coordinates were determined for the experimental dataset, radial shifts were found to be minor compared with the axial displacements; averaging only ~ 1.4% deviation between the M1 and R2 datasets. For clarity, only axial centroid shifts are therefore presented, as shown in Fig. 7. Repeat measurements at fixed conditions were used to estimate statistical uncertainty, calculated as ± 0.31 mm.

From Fig. 7, it can be observed that, irrespective of the insert used, flame length increases with rising thermal power and decreasing Φ. Additionally, increased surface roughness can be seen to produce a consistent axial shortening effect across all tested conditions, the magnitude of which scales with bulk velocity. This axial shortening is likely primarily driven by roughness-induced turbulence, particularly within the boundary layer (Vivoli et al. 2026). The effect becomes more pronounced at higher bulk flows, where the flame is more sensitive to turbulence-induced enhancements. In addition, enhanced wall heat loss under reacting hydrogen conditions is also known to increase quenching (penetration) distance near rough walls (Ding et al. 2021; Jaeschke et al. 2026; Zhu et al. 2026), and may contribute to the observed shortening by altering flame–flow interaction near the wall. Decoupling this thermal contribution from the turbulence-driven mixing enhancement discussed above would require near-wall flow and temperature diagnostics not available in the present dataset.

## Roughness Correlation Derivation

In this section, a novel *k*_*s*_ correlation is developed to provide accurate roughness modelling over the wide range of isothermal flows measured experimentally. The correlation proposed by Vivoli et al. (2025b) is first applied, followed by their derivation methodology. Having established a suitable correlation for the isothermal simulations, it was subsequently validated under reacting conditions.

### Application of Existing Correlations

Previous work by Vivoli et al. (2025b) proposed a *kₛ* correlation derived to guarantee a specified $\:{k}_{s}^{+}$ value in the fully rough regime. When applied to swirling flows in combination with a DES approach where ks^+^ > y^+^, the correlation was found to show good agreement under reacting CH₄ conditions. This correlation, referred to as FR-C (Fixed Roughness Correlation), is reproduced below for clarity.

1 $$\:\begin{array}{c}{k}_{s}\approx\:156Ra\end{array}$$

Given the R2 insert presented an average surface roughness of *Ra* = 21.2 μm, the FR-C correlation would require a *k*_*s*_ value of 3.31 mm - over 1/6 of the burner nozzle diameter - potentially overestimating experimental roughness effects. To address this, a *kₛ* value was derived for each isothermal test condition by following the methodology originally used by Vivoli et al. (2025b), referred to as GR-C (Guaranteed Roughness Correlation). The resulting *k*_*s*_ values for both FR-C and GR-C are collected in Table 5.

**Table 5:** k_s_ correlations calculated via GR-C and FR-C for initial test cases

	GR-C (mm)	FR-C (mm)
Iso_20	1.17	3.3072
Iso_32	0.76	
Iso_40	0.61	
Iso_64	0.41	

**Fig. 8:**
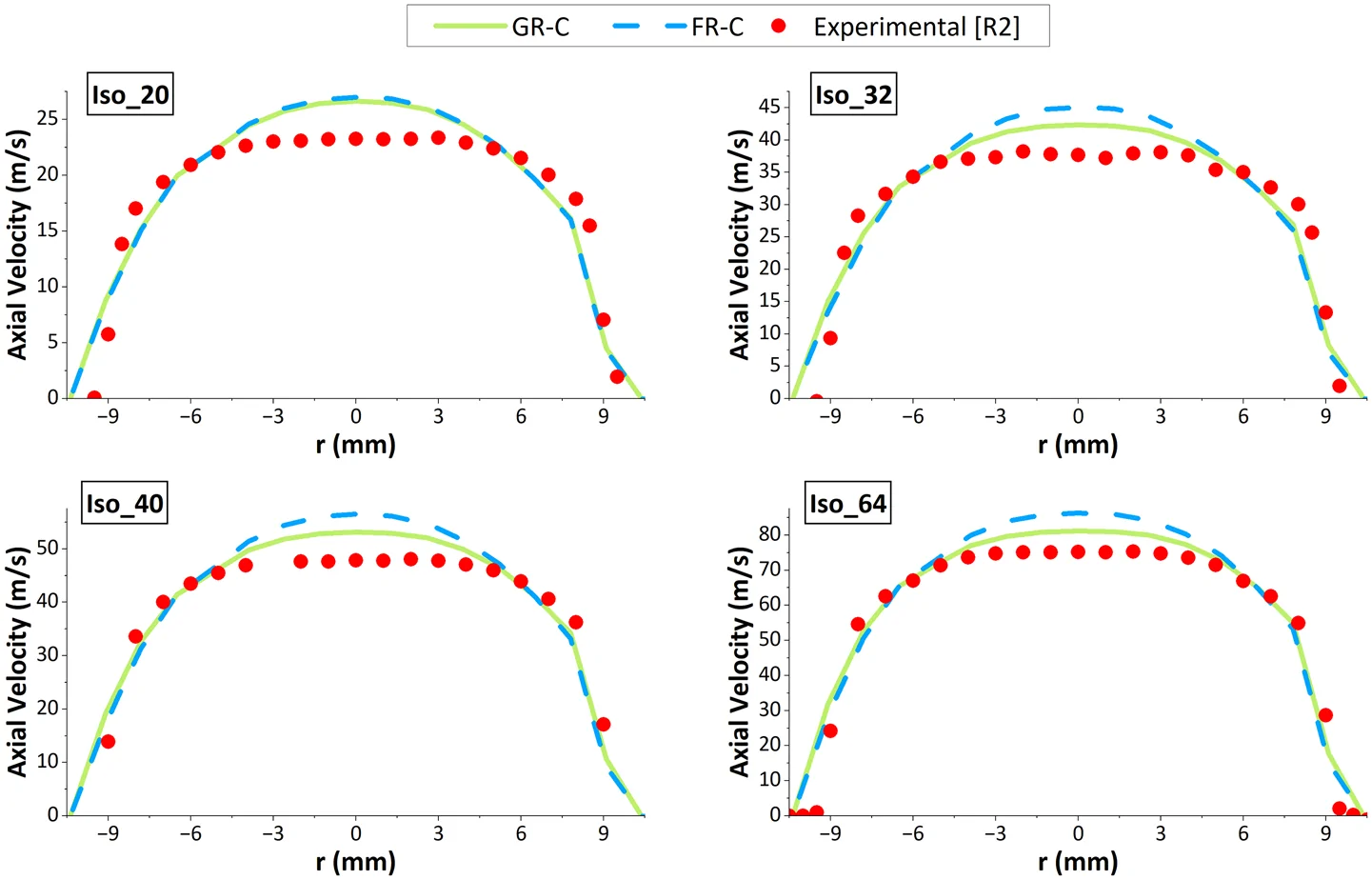
Comparison of numerical RANS rough velocity profiles generated with the GR-C and FR-C correlations compared against the R2 experimental data. Both datasets taken 2 mm above the nozzle exit

While the FR-C *k*_*s*_ value remains constant due to the fixed insert roughness, the GR-C values decrease with increasing bulk flow velocity as u* - proportional to the bulk velocity - is the only variable within *kₛ⁺* when under constant density and viscosity. Thus, the GR-C correlation depends not only on surface roughness height but also on bulk flow velocity. Initial rough RANS results with both the GR-C and FR-C correlations are shown in Fig. 8.

Figure 8 shows that roughness effects were overpredicted in all cases, with the discrepancy between numerical profiles increasing at higher bulk flows. For Iso_20, peak axial velocities differ by ~ 1.4%, while at higher flows the GR-C correlation predicts values ~ 6% lower than FR-C. Moreover, as bulk velocity increases, the GR-C predictions align more closely with experiments, with peak overprediction decreasing from ~ 14% (Iso_20) to ~ 7.8% (Iso_64).

From this initial assessment, neither the GR-C nor FR-C correlations provided satisfactory agreement with experimental data, although the GR-C correlation exhibited improved performance at higher bulk flow velocities. For the GR-C, the assumption of a constant $\:{k}_{s}^{+}$ proved inaccurate, since, with increasing bulk flows, the boundary layer thins, causing the fixed roughness elements to occupy a greater relative proportion of it resulting in a higher $\:{k}_{s}^{+}$ value (Kadivar et al. 2021; Schlichting 1979). In contrast, while the FR-C correlation predicts an increasing $\:{k}_{s}^{+}$with flow, the inconsistent offsets in peak numerical velocities relative to experiments highlight the need for an alternative modeling approach.

### Novel Correlation Derivation

**Fig. 9:**
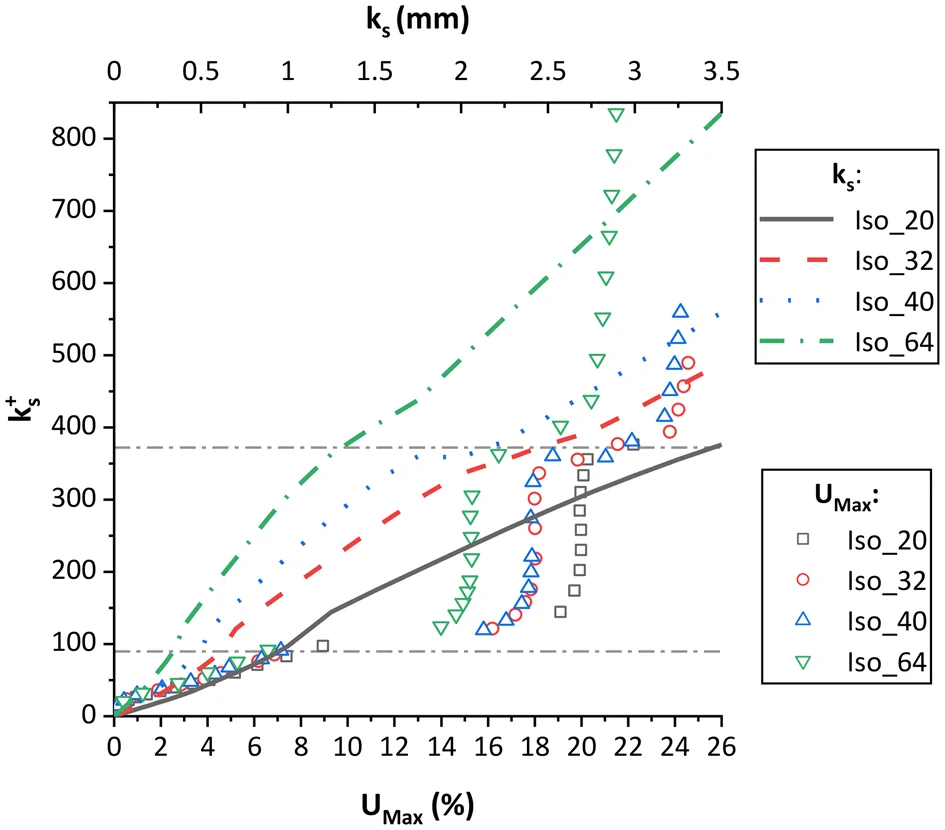
Changes in $\:{k}_{s}^{+}$ as a function of the applied k_s_ along with percentage changes in peak velocities at the nozzle exit relative to the reference smooth data. Reference lines set at both $\:{k}_{s}^{+}$ = 90 and $\:{k}_{s}^{+}$ = 372

Section 4.1 demonstrated that, for flow fields with a wide range of bulk flow velocities, neither fixing $\:{k}_{s}^{+}\:$nor using a correlation based solely on measured roughness height provided satisfactory results. Building on the findings from the GR-C, this section derives a new correlation that also incorporates bulk flow effects. The development of this correlation began with a parametric study conducted for the isothermal cases. A range of *k*_*s*_ values from 0 to 3.5 mm was applied to each RANS case, with each value being run for 1,000 iterations to ensure convergence before proceeding to the next roughness height. Both peak axial velocity and $\:{k}_{s}^{+}\:$values were monitored throughout. Changes in $\:{k}_{s}^{+}\:$as a function of applied *k*_*s*_, along with the corresponding percentage variations in peak velocity at the nozzle exit relative to a smooth reference are presented in Fig. 9.

From Fig. 9 it can be seen that $\:{k}_{s}^{+}$ values exhibit a near linear increase with increasing *k*_*s*_, regardless of the simulated bulk flow. The rate of increase of $\:{k}_{s}^{+}$ being proportional to the bulk flow for reasons already covered in Sect.  4.1. Focusing on the relative changes in peak axial velocities at the nozzle exit, values across all conditions exhibit a linear increase with increasing $\:{k}_{s}^{+}$ for $\:{k}_{s}^{+}$ < 90. However, within the range 90 ≤ $\:{k}_{s}^{+}$ < 150, a more than twofold increase is observed across all cases. Beyond this, velocity changes appear to plateau until another distinct jump occurs at $\:{k}_{s}^{+}$ ≈ 372. Following this jump, velocity changes asymptotically approach a limit, indicating a saturation effect. This occurs because, for $\:{k}_{s}^{+}$ ≥ 90, the flow is classified as being in the fully rough regime (Blocken et al. 2007). Consequently, alternative roughness function formulations are applied, as the sublayer becomes negligible due to the roughness elements exceeding its thickness (Blocken et al. 2007; Jayatilleke 1969).

With an understanding of the sensitivity of peak velocity to *k*_*s*_ values across various bulk flows, the focus shifted to determining the appropriate *k*_*s*_ required to replicate the experimental peak velocity shifts. Notably, for the bulk flows tested, the required *k*_*s*_ values all fell within the linear transitionally rough regime. To obtain an exact *k*_*s*_, data fitting was applied to each dataset, generating equations where peak axial velocity served as the *x*-value and *k*_*s*_ as the *y*-value. By inputting the experimentally measured peak axial velocity into these equations, the corresponding *k*_*s*_ value was determined.

The *k*_*s*_ values extracted demonstrated that, as the bulk flow increases, progressively smaller values are required to replicate the roughness effects observed experimentally, similar to the behavior seen with the GR-C in Sect.  4.1. However, unlike for the GR-C, each *k*_*s*_ value in this section corresponds to a significantly different $\:{k}_{s}^{+}$ value, addressing the limitations of the GR-C approach.

To develop a generalized correlation, the $\:{k}_{s}$ values derived for individual bulk flows were plotted against bulk velocity. Non-linear regression was applied to capture the tapering of $\:{k}_{s}$ at higher flows, consistent with the reduced roughness sensitivity for $\:{k}_{s}^{+}>90$ observed in Fig. 9. However, since the polynomial alone predicts increasing $\:{k}_{s}$ beyond its vertex, contradicting the saturation trend, a piecewise formulation was adopted, applying the quadratic fit up to the vertex and a fixed linear value thereafter. To account for measured roughness effects, the roughness parameter was incorporated into the constant coefficients. The final correlation is presented in Eq. (2).

2 $$\:\begin{array}{c}{k}_{s}\left(x\right)=\left\{\begin{array}{c}0.0945{x}^{2}-14.68x\:+41.17Ra,\:\:x\le\:77\\\:41.17Ra\:-570\:\:\:\:\:\:\:\:\:\:\:\:\:\:\:\:\:\:\:\:\:,\:\:x>77\end{array}\right.\end{array}$$

Where:

*x* = Bulk flow velocity (m/s).

Ra and *k*_*s*_ are in µm.

The above correlation was validated under atmospheric isothermal-air conditions for bulk flow velocities (*x*) between 20 and 64 m/s within the PJB burner architecture as outlined in Sect.  5. The polynomial segment of Eq. (2) was obtained via least-squares regression against the experimental dataset (R² = 0.993, valid for 20 ≤ *x* ≤ 64), while the linear segment is a prescribed form used to conservatively extend the correlation trend beyond the polynomial vertex (*x* ≈ 77.7 m/s) and is not itself fitted to experimental data. For bulk flows outside this range, and for differing burner geometries, the *k*_*s*_ values obtained via Eq. (2) should be treated as an initial approximation rather than a validated prediction. Its applicability under reacting atmospheric hydrogen conditions is assessed in Sect.  6.

## Isothermal Validation

### RANS

**Fig. 10:**
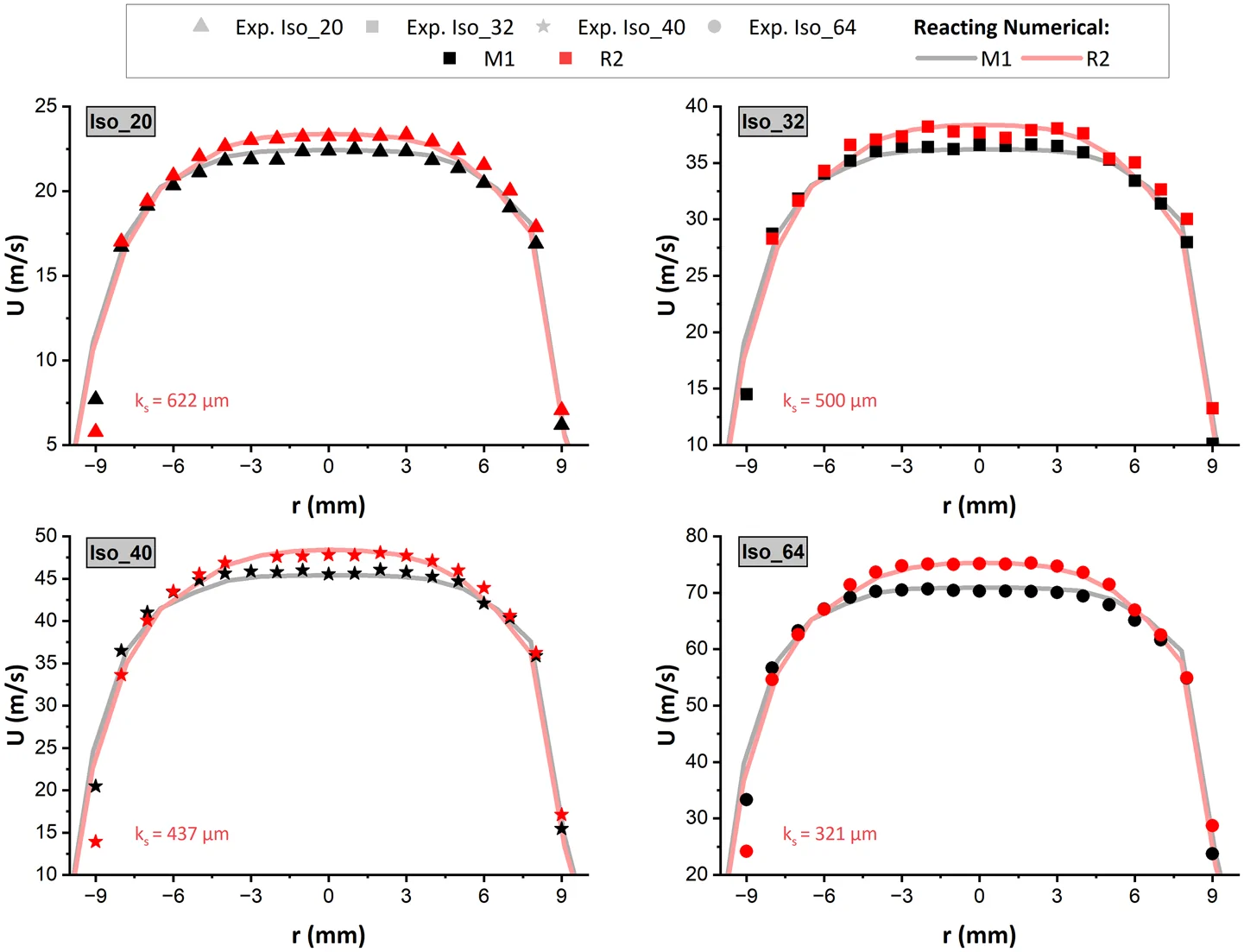
Comparison of experimental and numerical (RANS) smooth and rough axial velocity profiles 2 mm above nozzle exit. Squares in legend indicating the color code

Following the development of the novel *k*_*s*_ correlation, the bulk flows for the Iso 20, 32, 40 and 64 cases were input into Eq. (2) together with the measured Ra value of the R2 insert. The corresponding *k*_*s*_ values were then implemented in RANS simulations for each condition. The resulting velocity profiles were systematically evaluated against reference smooth simulations as well as experimental velocity profiles, as presented in Fig. 10.

Visual inspection of Fig. 10 shows that using the $\:{k}_{s}$values from Eq. (2) yields strong agreement with experimental rough-wall data across all bulk flows. Quantitatively, smooth numerical profiles deviate from experiments by ~ 1.36% across the full span (− 9 mm ≤ *r* ≤ 9 mm) and ~ 0.14% in the core (− 5 mm ≤ *r* ≤ 5 mm). Rough-wall profiles show peak deviations of ~ 0.5%, with an average profile deviation of ~ 2.4% overall and ~ 0.4% within the core. Overall, both smooth and rough numerical datasets can be seen to present excellent agreement with the experimental data.

### DES

**Fig. 11:**
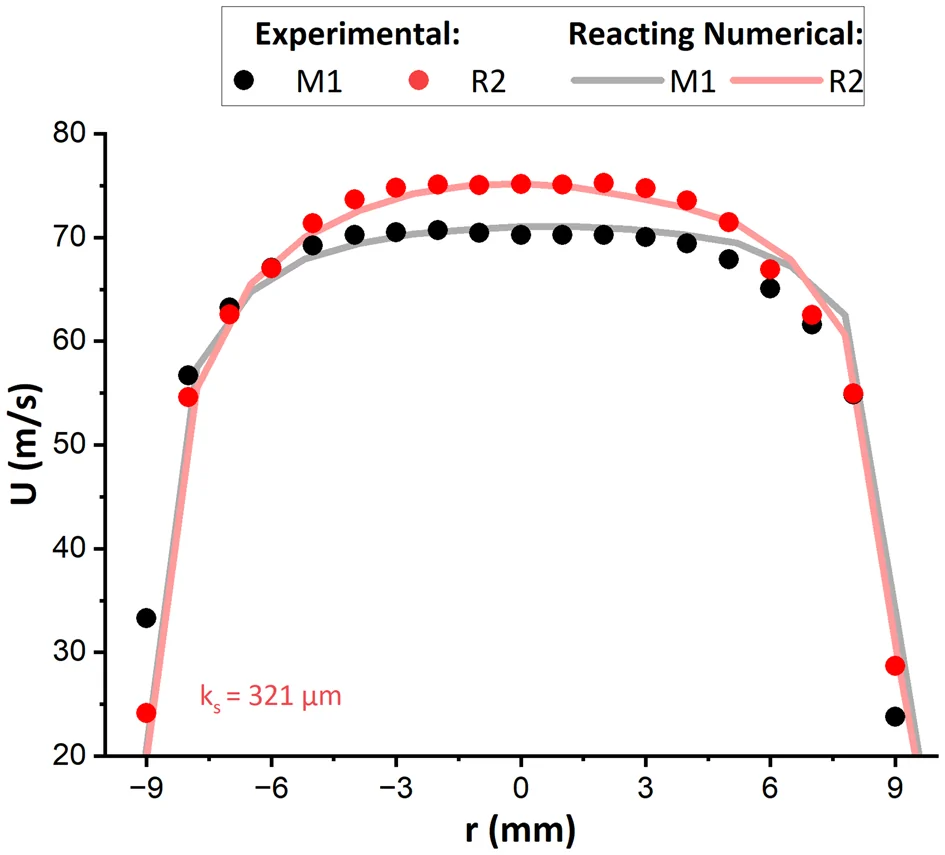
Comparison of Iso_64 experimental and numerical (DES) smooth and rough axial velocity profiles 2 mm above nozzle exit

As previously covered in Sect.  2.2.1, due to the computational demand of DES, the effectiveness of the novel roughness correlation was only assessed on the Iso_64 case. For the rough case, the same *k*_*s*_ value utilized for Iso_64 in Sect.  5.1 was applied. Numerical results and the respective experimental counterparts are presented in Fig. 11.

Figure 11 demonstrates that numerical results closely matched experiments, consistent with RANS observations. When compared with the respective experimental datasets, peak numerical velocity deviations were found to be ~ 0.2%, with average radial deviations of ~ 0.88%, and core flow deviations of ~ 0.14%.

## Reacting Validation

Building on the efficacy demonstrated by the novel correlation when applied to isothermal PJB cases, its applicability to reacting flow simulations was further investigated using both RANS and DES approaches. The specific *k*_*s*_ values utilized for each condition are summarized in Table 6.

**Table 6:** k_s_ values utilized in the reacting simulations calculated using Eq. (2)

Thermal Power (kW)	φ	k_s_ (µm)
25	0.9	478
	0.7	423
	0.5	353
20	0.9	536
	0.7	489
	0.5	410
15	0.9	606
	0.7	565
	0.5	495

### RANS

#### Flow Characterization

**Fig. 12:**
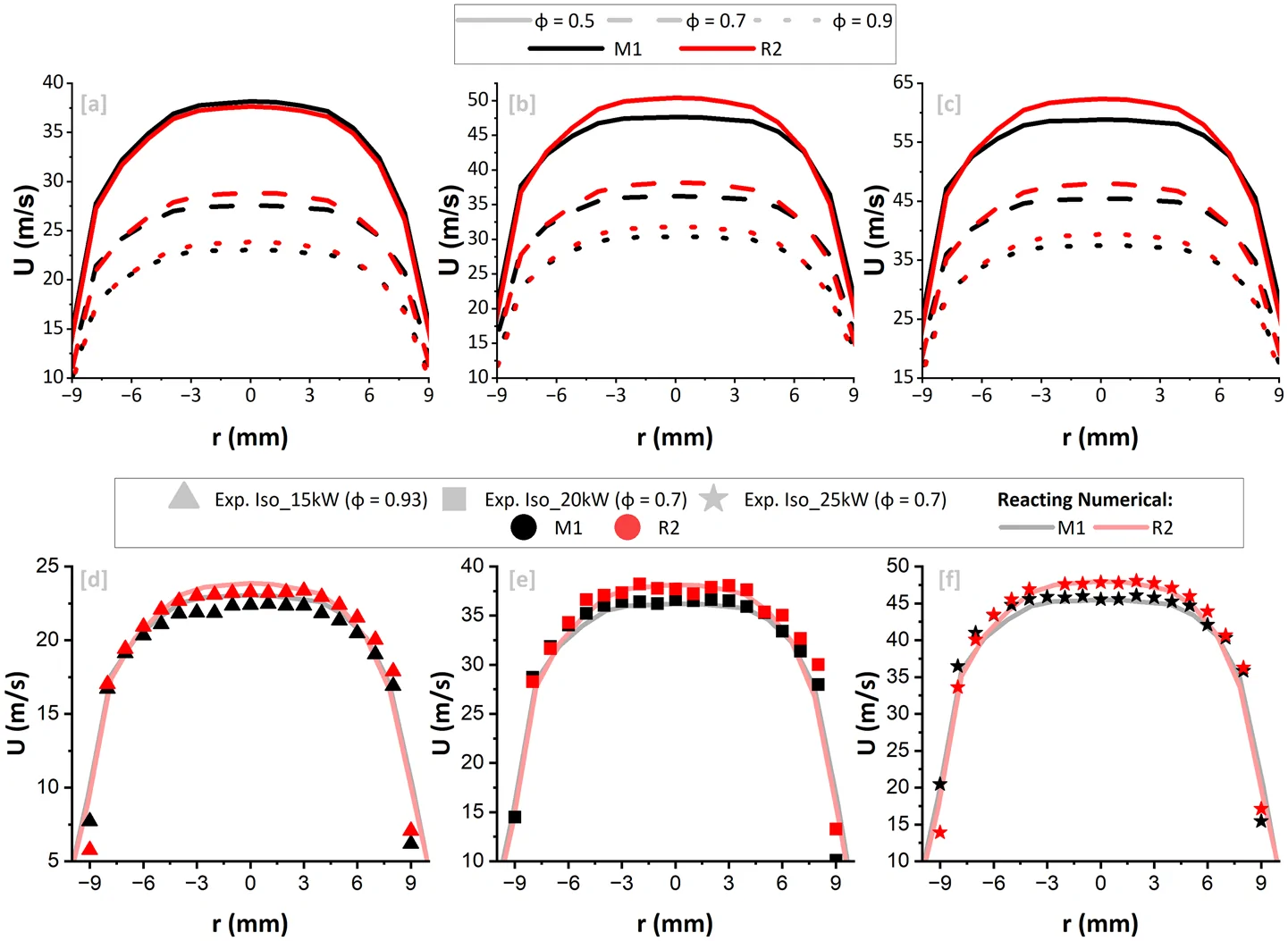
Comparison of reacting numerical (RANS) smooth and rough axial velocity profiles 2 mm above the nozzle exit with φ = 0.5–0.7–0.9 at thermal powers of 15 kW [**a**], 20 kW [**b**] and 25 kW [**c**]. Comparison of isothermal experimental and reacting numerical (RANS) smooth and rough axial velocity profiles 2 mm above the nozzle exit [**d**, **e**, **f**]. Circles in legend indicating the color code

As noted at the outset of this work, experimental flow-field measurements were conducted exclusively under isothermal conditions. Although direct comparison with numerical reacting flows is not strictly representative, thermal effects are expected to be minimal at 2 mm downstream of the nozzle, where inertial dynamics dominate. Yet, wall-to-flow temperature differences become more significant than under isothermal conditions. In addition to the turbulence-enhancement mechanism discussed in Sect.  3, roughness-induced changes in wall heat flux (Ding et al. 2021; Jaeschke et al. 2026; Zhu et al. 2026), and the associated local density and viscosity effects, represent a potential additional mechanism contributing to the observed velocity redistribution. As this thermal-coupling effect is not resolved in the present simulations (Sect.  2.2), the reacting-flow results presented here capture only the turbulence-driven contribution to roughness-induced flow redistribution. Nevertheless, comparison of the isothermal measurements with the reacting simulations remains useful for assessing general flow trends. Figure 12 presents axial velocity profiles for the reacting smooth- and rough-wall cases together with the isothermal experimental data.

Figure 12 [a-c] shows that peak axial velocities increase with thermal power and decreasing φ. Except for the 15-kW case at φ = 0.5, all rough-wall simulations exhibit higher peak centerline velocities than smooth-wall cases, replicating experimental trends. Mean bulk flow velocities varied by only ~ 1.6% on average, indicating a roughness-induced redistribution of the profiles. Comparison with experimental isothermal data (Fig. 12 [d-f]) shows similarly strong agreement. Exact matches are not expected due to thermal effects with average deviations across the full radial profiles of ~ 7.5%, 3.0%, and 1.3% for 15-, 20-, and 25-kW, respectively. The numerical offset decreasing with increasing power due to stronger inertial forces and lower equivalence ratios reducing thermal influence.

#### Flame Behavior

Flame centroid locations were extracted for both experiments and simulations to quantify the ability of the numerical model to reproduce roughness-induced changes in flame shape, as described in Sect.  2.1.2. Experimental OH^*^ CL is a line-of-sight-integrated signal, summing emission through the full depth of the turbulent flame brush and over the imaging exposure time, which produces a broad, continuous intensity profile well-suited to stable centroid calculation. Numerical OH^*^ fields, by contrast, are extracted from a single computational plane and therefore represent only the thin reaction-zone location at that plane, without the line-of-sight averaging present in the experimental measurement; this produces a filamentary field, with the overall volume sensitive to local intensity fluctuations, making direct centroid extraction unreliable.

**Fig. 13:**
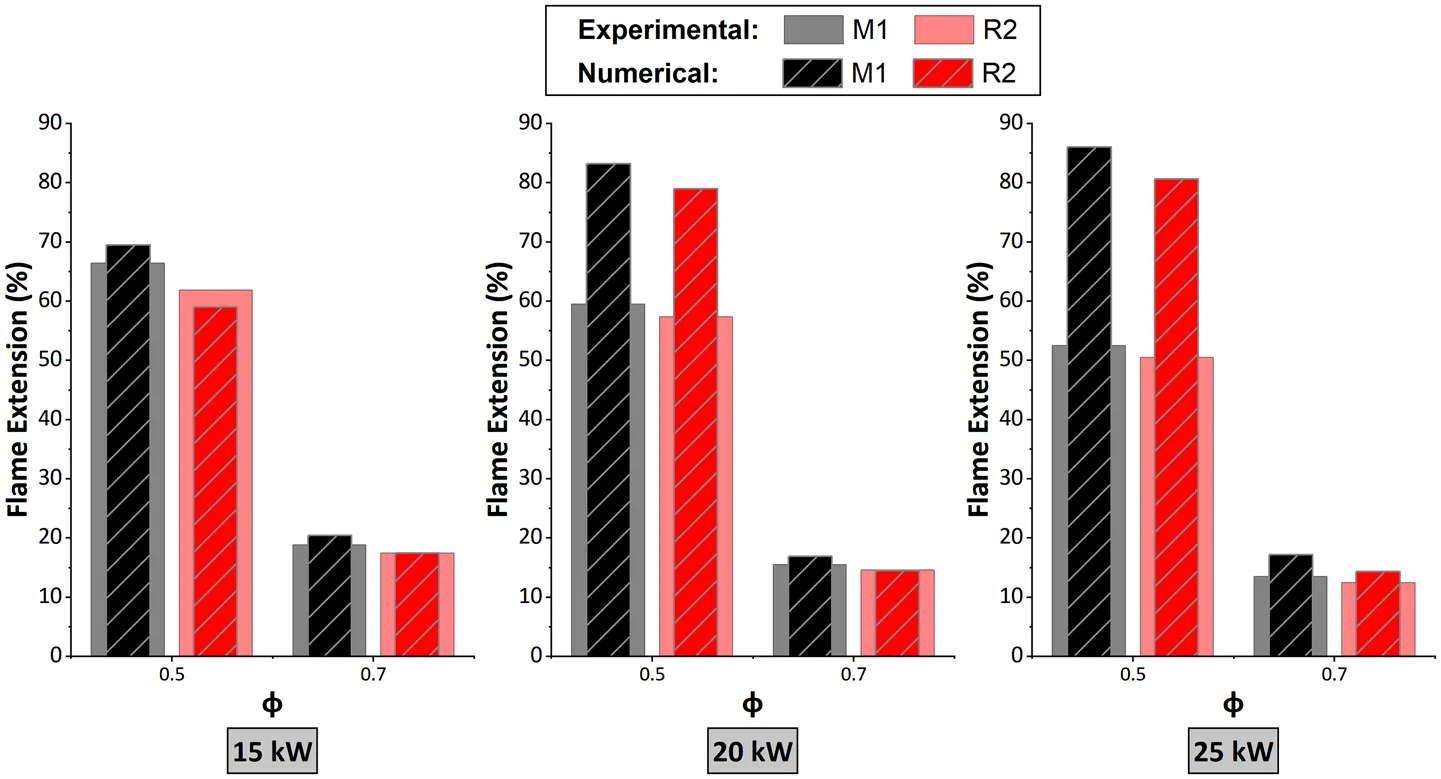
Changes in experimental and numerical (RANS) flame centroid location. Values have been normalized relative to the corresponding φ = 0.9 cases

To address this, numerical centroid data were instead computed from the spatial distribution of unreacted H_2_ within the flame region. Since fuel is consumed at the flame front where OH^*^ is produced, the extent and shape of the unreacted-fuel field and the OH^*^ field trace the same underlying flame envelope from opposite sides. This equivalence is supported qualitatively in Fig. S6 (Supplementary Material), which shows close agreement in the shape and axial extent of the normalised H₂ mole fraction and OH* fields. The unreacted-fuel-based centroid therefore provides a numerically robust and physically consistent basis for comparison with the experimental OH*-derived centroid, while avoiding the sensitivity to local fluctuations inherent to the filamentary numerical OH* field. In order to isolate the discrepancies in magnitude between experimental and numerical data, centroid variations were normalized relative to the respective φ = 0.9 cases. The resulting trends are shown in Fig. 13.

Figure 13 shows that while simulations capture the trend of axial flame lengthening with decreasing φ, they increasingly overpredict this effect at higher bulk flows. The 15 kW cases deviate by only ~ 2.2% from experiments, whereas deviations rise to ~ 21.3% and ~ 41.3% for 20 kW and 25 kW, respectively. For 20 kW, φ = 0.7 deviates ~ 3.8%, but φ = 0.5 rises to ~ 38.8%; similar but larger deviations occur at 25 kW. These results indicate that above ~ 33 m/s, the models fail to capture hydrogen diffusion accurately, causing overpredicted flame lengths. This growing discrepancy at higher thermal power and leaner conditions is consistent with the increasing sensitivity of lean hydrogen flames to the modelling simplifications discussed in Sect.  2.2.2. In addition, given wall heat flux was not directly accounted for, this likely underpredicts quenching distances and could compound the overprediction of flame length.

From a roughness standpoint, Fig. 13 shows that, consistent with experimental observations, roughness-induced axial flame shortening - represented by the offset between flame extensions at each condition - is captured numerically across all conditions considered, with the magnitude increasing at higher bulk flow rates (i.e., lower φ and higher thermal power). However, the simulations tend to overpredict the extent of axial shortening, particularly at lower thermal powers. The mean experimental-to-numerical deviations in shortening magnitude being approximately 19.2%, 230%, and 271% for 25 kW, 20 kW, and 15 kW, respectively, with the largest discrepancy observed at 15 kW and φ = 0.5, corresponding to the highest kₛ value. Although increased kₛ at lower thermal powers likely contributes to this overprediction, the absence of a consistent correlation across all cases indicates that additional mechanisms, including changes in heat flux, also influence the roughness effect.

### DES

As covered in Sect.  2.2.2, due to the computational demand of DES, the effectiveness of the novel roughness correlation was only assessed on the 25 kW cases. Similar analyses and methodologies employed for the reacting RANS results are applied in the following subsections.

#### Flow Characterization

**Fig. 14:**
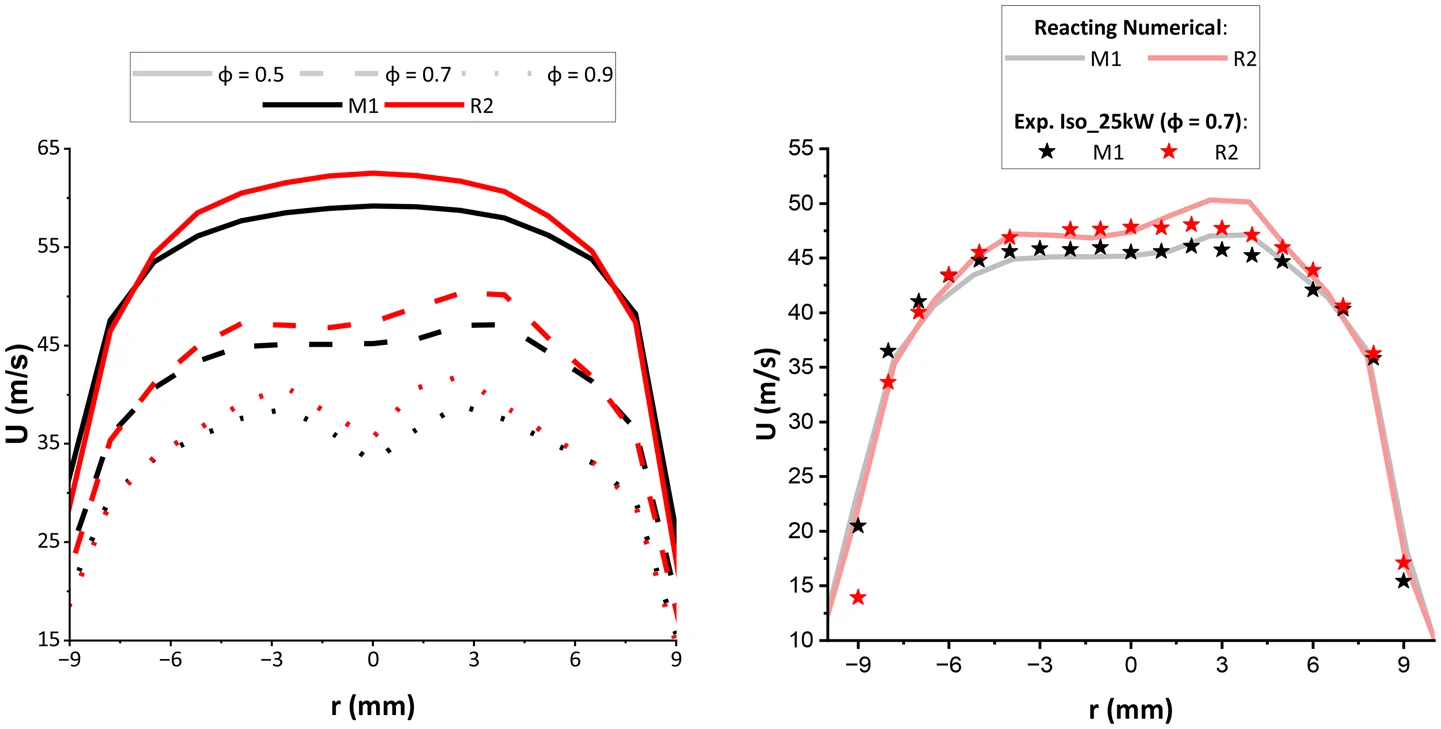
Comparison of reacting DES smooth and rough axial velocity profiles at 25 kW (φ = 0.5 and 0.9), 2 mm above the nozzle exit [left], and corresponding isothermal experimental vs. reacting DES profiles [right]

Although experimental measurements were isothermal, reacting RANS results showed reasonable agreement. Accordingly, Fig. 14 presents the axial velocity profiles from the reacting DES simulations, extracted 2 mm downstream of the nozzle exit, alongside comparisons with the reference isothermal experimental data.

From Fig. 14, peak axial velocities increase with rising thermal power and φ, as expected. Moreover, all rough-wall simulations exhibit higher peak centerline velocities compared to their smooth-wall counterparts, effectively replicating the trends observed experimentally. It is also evident that, as bulk flow rates decrease, both smooth- and rough-wall velocity profiles increasingly diverge from the characteristic parabolic shape, previously observed experimentally and in the RANS simulations, and instead assume a more pronounced M-shaped profile. This deviation is attributed to the DES model overestimating the influence of the central bluff body (Pereira et al. 2018, 2019), a behavior previously identified in other works (Vivoli et al. 2025b).

In addition, asymmetry is particularly noticeable in the φ = 0.7 case. This may be attributed to initial instabilities at the start of the simulation that were not fully eliminated before time averaging, as well as other factors such as limited grid resolution. Nevertheless, when comparing the DES axial velocity profiles with the corresponding RANS and isothermal experimental core-flow data (*r* ± 5 mm), the deviations remain within low single-digit percentages across all cases, ranging from approximately 0.4% for φ = 0.5 to ~ 3.7% for φ = 0.9.

#### Flame Behavior

**Fig. 15:**
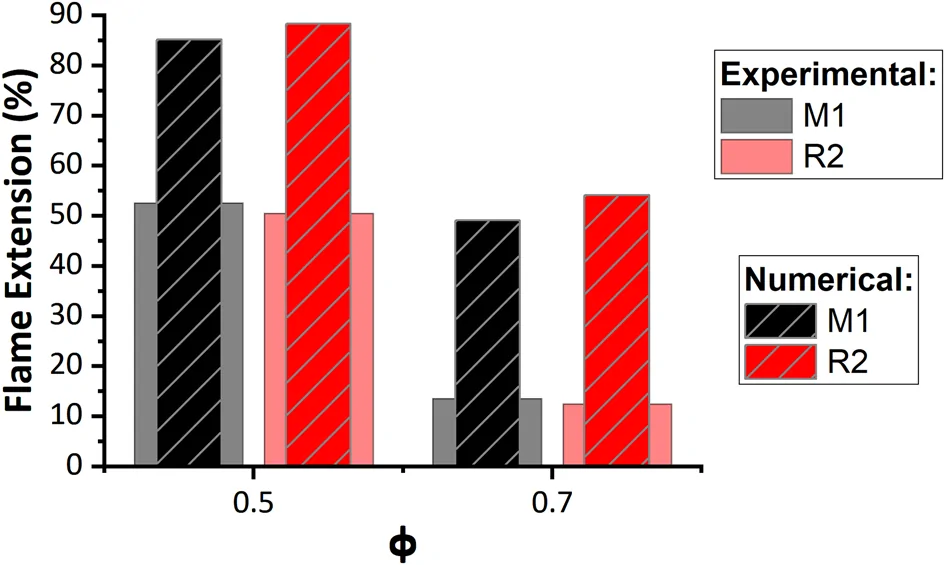
Changes in experimental and numerical (DES) flame centroid location. Values have been normalized relative to the corresponding φ = 0.9 cases

**Fig. 16:**
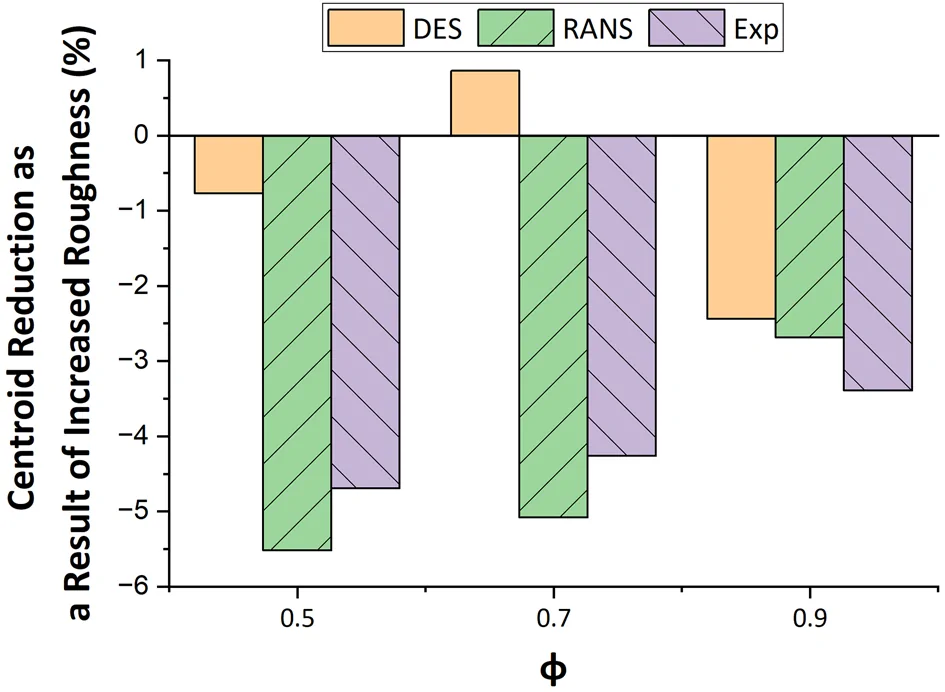
Reduction in the axial coordinate of 25 kW numerical and experimental centroids as a function of roughness. Values have been normalized relative to the corresponding smooth-wall cases

As per Sect.  6.1.2, assessment of roughness induced axial shortening was performed by computing the flame centroid location for each case. With centroid variations normalized both relative to the φ = 0.9 cases and based on roughness. The resulting DES trends are shown in Figs. 15 and 16 respectively.

Figure 15, shows that DES captures the trend of flame lengthening with decreasing φ but deviates from RANS results. At φ = 0.5, DES and RANS flame lengths agree (within ~ 1%), whereas at φ = 0.7, DES overpredicts axial extension by ~ 65%. Additionally, the magnitude of roughness induced flame shortening is significantly dampened at lower φ, as highlighted by the greater relative flame lengthening in Fig. 15, opposite to the experimental. This indicates that, whilst DES captures roughness effects, the magnitude of flame shortening is less pronounced, as shown in Fig. 16.

Figure 16 shows that, for φ = 0.9, DES captures a clear roughness-induced offset, deviating only ~ 10% from RANS. For φ = 0.5 and 0.7, roughness effects are minimal (± 0.9%), failing to reproduce the larger offsets seen experimentally and in RANS, with an inverse trend at φ = 0.7. Overall, while DES captures roughness effects in the flow field, their impact on flame composition is limited, likely due to complex turbulence-chemistry interactions and the limited transfer of wall-function-based roughness models to chemical response, especially at lower φ where hydrogen thermodiffusive effects are significant (Fruzza et al. 2024, 2025). In addition, by resolving a greater fraction of the near-wall turbulent structures, DES could be more sensitive to inaccuracies in the prescribed wall thermal boundary condition than RANS, where such errors are partially absorbed by the turbulence closure rather than propagated directly into the resolved flame response.

### Changes in *k*_*s*_^+^

An analysis of changes in *kₛ⁺* values between reacting and isothermal cases revealed that, under equivalent conditions, the reacting cases exhibited an average reduction of ~ 21.6% in *kₛ⁺* compared to the corresponding isothermal air cases with equivalent total mass flow. From Eq. (3), this consistent reduction across all cases can be attributed to a decrease in fluid density (ρ) resulting from the introduction of hydrogen.

3 $$\:\begin{array}{c}{k}_{s}^{+}=\frac{{k}_{s}\:{u}^{*}\rho\:}{\mu\:}\end{array}$$

Under equivalent conditions, *kₛ* is constant and u* varies minimally (~ 1.7%) between isothermal and reacting simulations. However, hydrogen reduces ρ by ~ 22% and dynamic viscosity (µ) by ~ 1.4%. As a result, although *kₛ⁺* is lower in reacting cases, roughness effects can be more pronounced due to increased sensitivity of reacting flows to wall-induced turbulence and turbulence-chemistry interactions. This underscores that while *kₛ⁺* remains a useful metric, its interpretation in reacting flows, especially under elevated combustor pressures or varying fuel properties, requires caution, as matching *kₛ⁺* across conditions may produce physically inconsistent comparisons.

## Conclusions

A range of approaches were investigated to numerically replicate the experimentally observed roughness effects under both isothermal and reacting conditions, using resolved boundary layer (y^+^ ≈ 1) meshes in RANS and DES frameworks. The correlation originally derived by Vivoli et al. (2025b) was first applied but consistently overpredicted roughness effects. Building on the same methodological framework, a novel roughness correlation was developed that incorporated both bulk flow and measured surface roughness as parameters.

Application of the new correlation under isothermal conditions demonstrated both RANS and DES simulations exhibited high accuracy. RANS cases presenting low single-digit percentage deviations from experimental data, validating the effectiveness of the correlation while offering computational efficiency. With DES further improving accuracy albeit at increased computational cost.

Extending the correlation to reacting conditions, RANS results again showed good agreement with experimental observations, capturing key roughness-induced effects on the flow field and flame structure. By contrast, DES performance was less satisfactory, with persistent overprediction of bluff-body effects and limited capability in reproducing roughness-driven changes in flame shape, especially under lean conditions.

In light of these findings, the following conclusions are drawn:

- For datasets spanning a wide range of bulk flow conditions (i.e., varying Reynolds numbers), roughness correlations based solely on measured roughness height (kₛ) are insufficient to accurately represent roughness effects across the entire parameter space. Instead, correlations incorporating an additional flow-dependent parameter are required.
- Accurately modelling roughness-induced effects on flame composition is considerably more challenging. Even when flow-field modifications are well captured, corresponding changes in flame shape or centroid location are not guaranteed. Significant differences in sensitivity are observed between RANS and DES approaches, with complex turbulence–chemistry interactions likely contributing; the influence of alternative chemical mechanisms and thermal effects remains an open question.
- Reacting cases were frequently found to overpredict roughness effects at a given kₛ, despite density-driven reductions in kₛ⁺. This highlights the need for increased attention when dealing with inlet conditions where changes in temperature, fuel composition, and other operating parameters can substantially modify density and consequently kₛ⁺ compared to reference cases.

In summary, the novel roughness correlation demonstrated strong capability to replicate experimental roughness-induced trends under isothermal conditions. Under reacting-flow conditions, the complexities of near-wall premixed flame dynamics - including wall heat transfer and its associated effect on quenching distance - proved more challenging to capture accurately in the fluid-only simulations performed here, particularly with higher-fidelity DES. Despite these limitations, the correlation offers a practical and effective tool for industry, provided its current constraints are recognized, and serves as a validated baseline that can be further developed to more accurately capture coupled thermal and flow field interactions under reacting conditions.

## Supplementary Information

Below is the link to the electronic supplementary material.

Supplementary Material 1

## Data Availability

Information on the data underpinning this publication, including access details, can be found in the Cardiff University Research Data Repository at [https://doi.org/10.17035/cardiff.30464906].
