# Supplementary material for: Novel Low-y⁺ Roughness Correlation for Hydrogen-Fired Jet Burners Validated Under Isothermal and Reacting Conditions
Source: Flow Turbul Combust. 2026 Oct 6;117(3):40. doi: 10.1007/s10494-026-00799-z (PMC13642420; doi:10.1007/s10494-026-00799-z)
Supplement: Supplementary file 1 [file 10494_2026_799_MOESM1_ESM.pdf]

Dr. Robin Vivoli - Supplementary data for Novel Low-y^+ Roughness Correlation for Hydrogen-Fired Jet Burners Validated under Isothermal and Reacting Conditions – Flow, Turbulence and Combustion

This supplementary document presents additional data and methodologies referenced throughout the paper. During experimental operation, thermocouple measurements were obtained at three locations: within the bluff-body holder (T_{Inlet}), at the outlet of the quartz confinement (T_{EG}) and at the base of the combustion chamber (T_{CC}). These data were used to define boundary conditions for numerical simulations. Fuel and air inlet conditions, along with corresponding thermocouple readings, are summarized in Table S1. The reference pressure was set to atmospheric (0.101 MPa). Mesh dependency data for both isothermal and reacting conditions are presented in Figures S1-S3. Velocity profile developments under isothermal conditions for a combustion chamber with 10x expansion are presented in Figure S4. These data were utilized to inform sizing of the expansion ratio of the isothermal domain utilized within the paper. Visualization of the premixing level within the PJB obtained via an exploratory RANS simulation of the entire burner system is shown in Figure S5. Figure S6 shows the spatial distribution of unburnt H_2 upstream of the flame front with the corresponding normalized OH* field overlayed, each normalized independently to its maximum value within the domain. This figure supports the equivalence of the unburnt-H_2 and OH*-based flame envelopes, justifying the use of the unburnt H_2 region for numerical flame centroid extraction, as discussed in Section 6.1.2.

Table S1 *- Inlet and outlet conditions for numerical reacting simulations.*

Thermal Power (kW)	φ	$\dot{m}_{fuel/air}$ (g/s)	Mass Fraction			T_{Inlet} (K)	T_{CC} (K)	T_{EG} (K)
			H_2	O_2	N_2			
**25**	0.9	8.13	0.026	0.227	0.747	284	707	1510
	0.7	10.47	0.020	0.228	0.752		683	1409
	0.5	14.46	0.014	0.230	0.756		685	1290
**20**	0.9	6.59	0.025	0.227	0.748		668	1472
	0.7	8.27	0.020	0.228	0.752		500	1372
	0.5	11.63	0.014	0.230	0.756		622	1262
**15**	0.9	4.95	0.025	0.227	0.748		668	1417
	0.7	6.25	0.020	0.228	0.752		635	1337
	0.5	8.61	0.015	0.230	0.756		634	1237

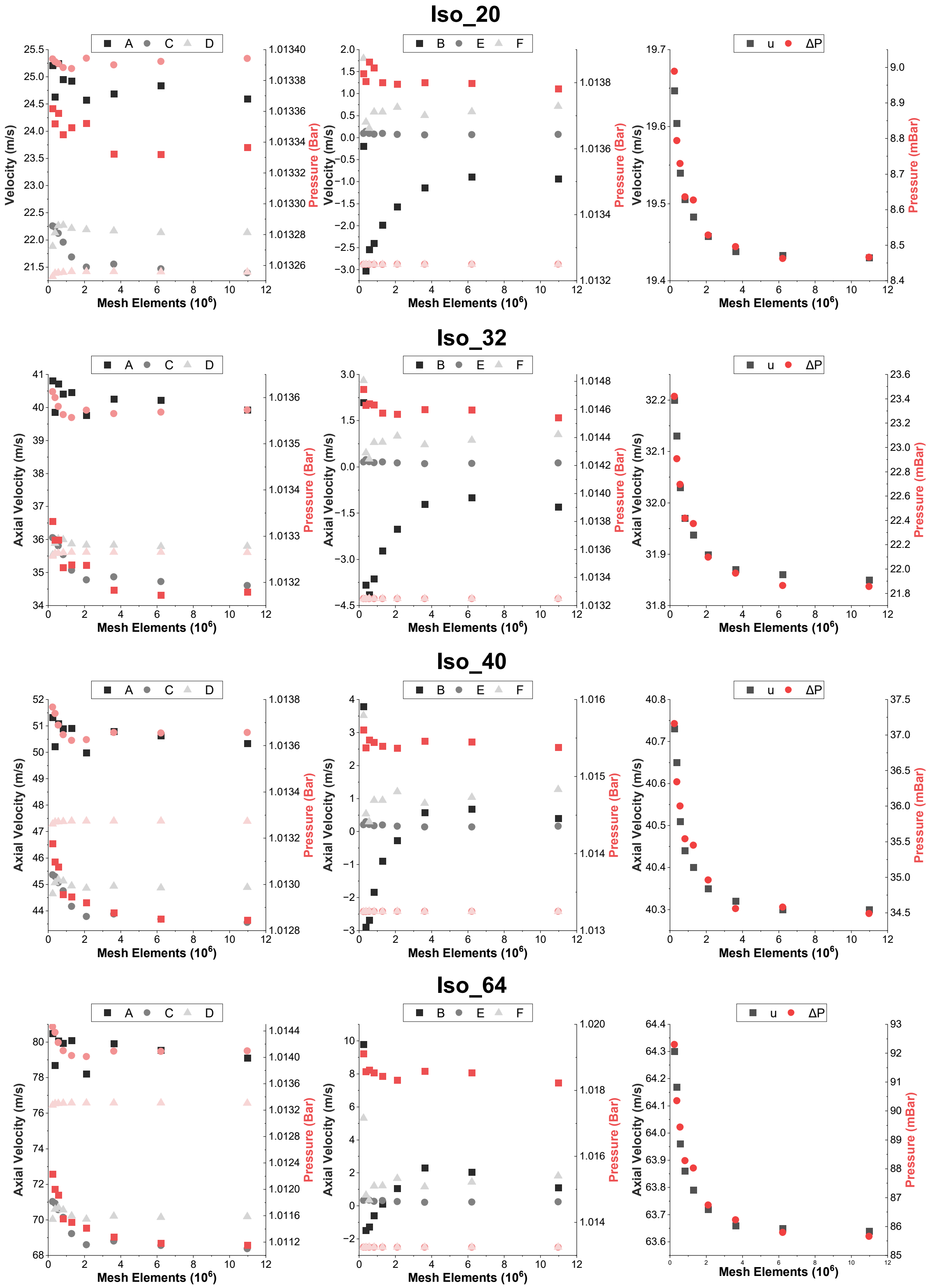

Figure S1 – Isothermal RANS mesh dependency results for Iso_20, Iso_32, Iso_40 and Iso_64 simulations. See Figure S2 for point probe locations.

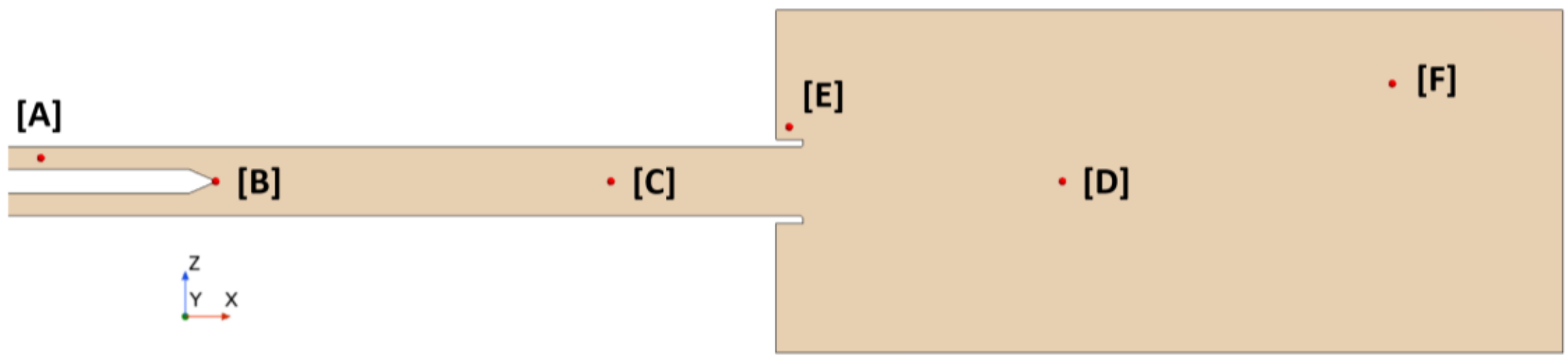

Figure S2 - *Point Probe location for mesh dependency tests associated with Figure 1.*

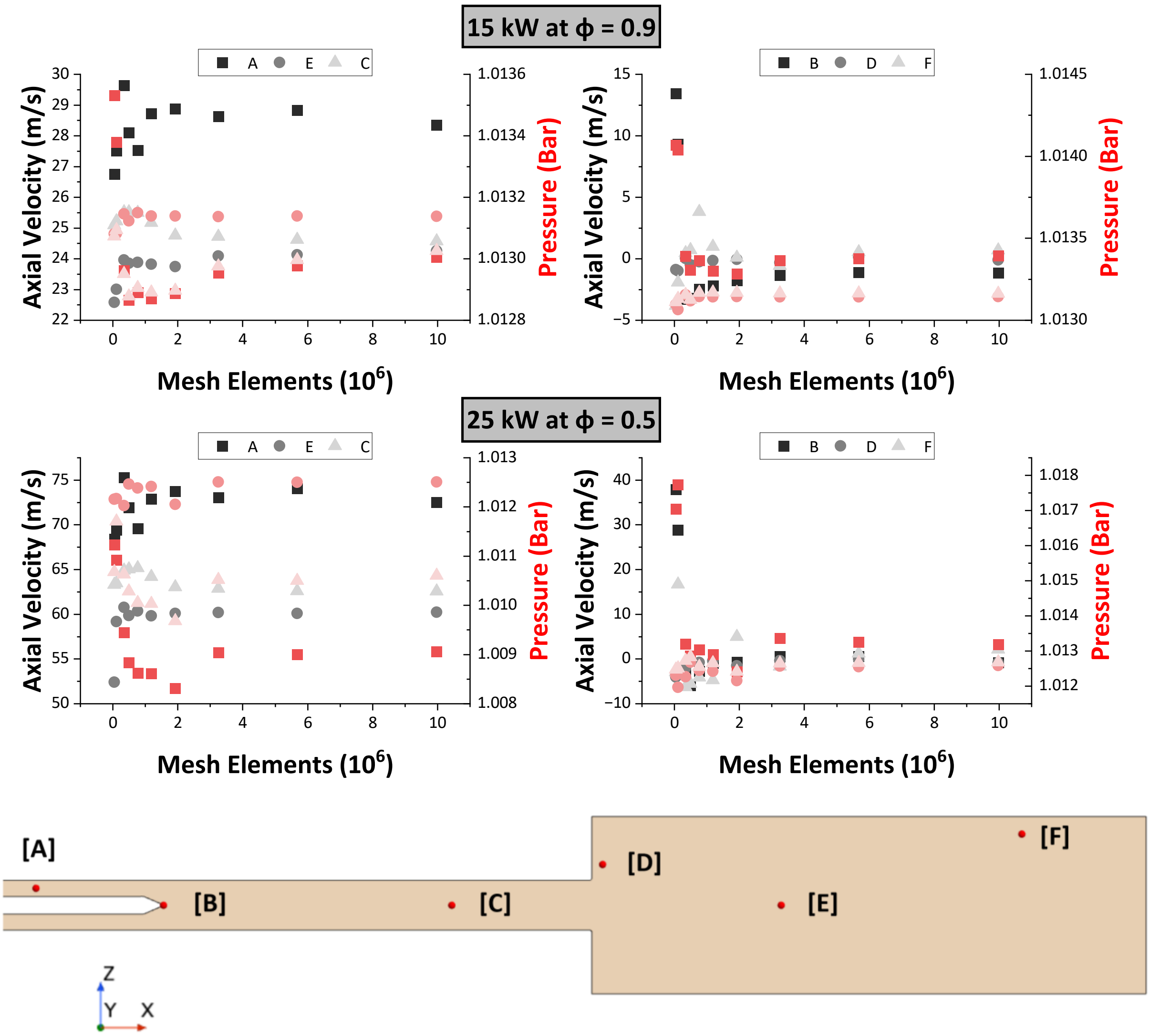

Figure S3 - *Mesh dependency results for the most extreme reacting RANS cases [top]. Point probe locations [bottom].*

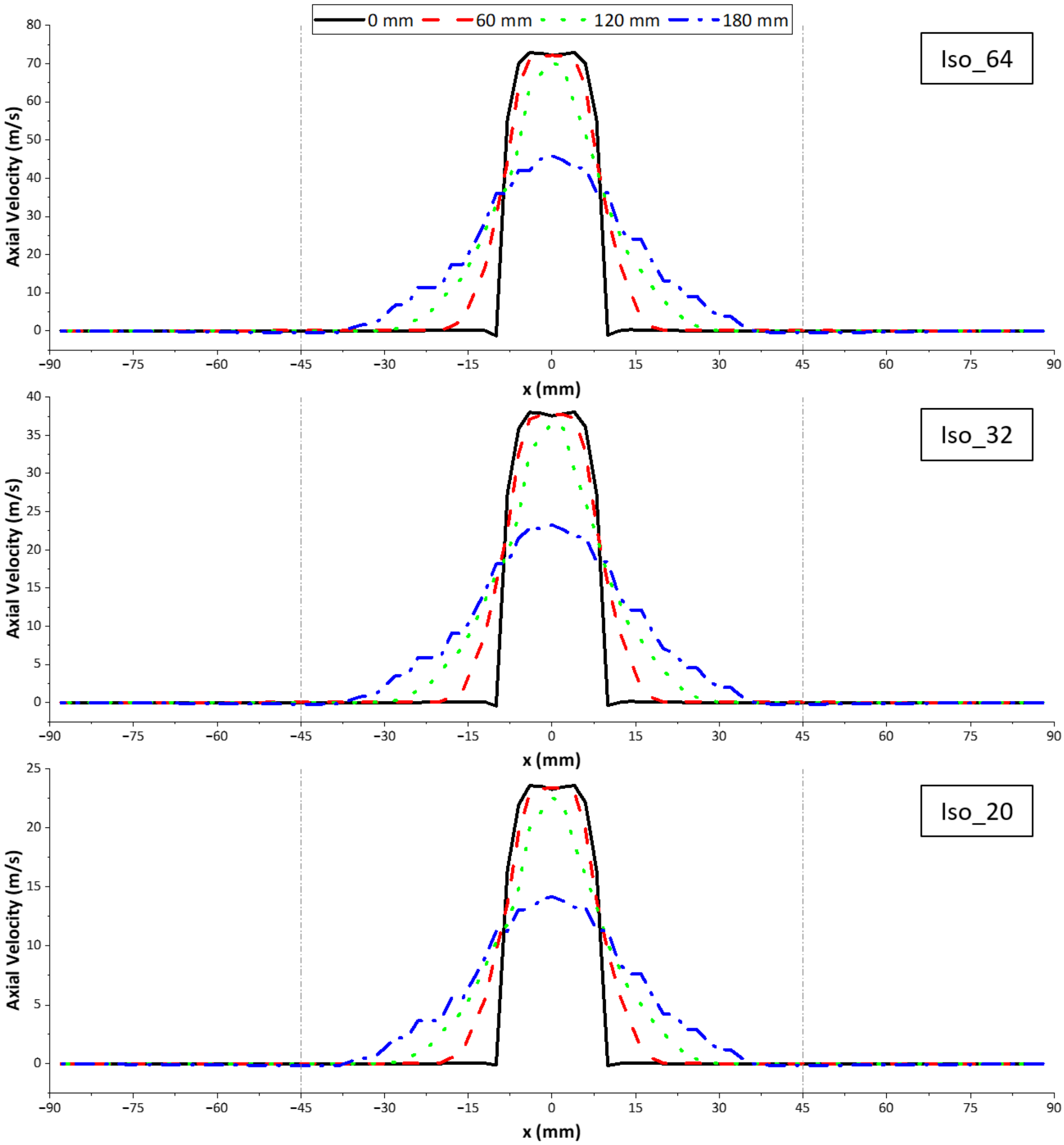

Figure S4 – Isothermal RANS axial velocity profile development across the unconfined combustion chamber with 10x expansion. Line probes placed at 60mm intervals covering the entire combustion chamber starting from 2mm above the nozzle exit.

Note how for x values > 45 axial velocity values ≈ 0 m/s for all probe locations

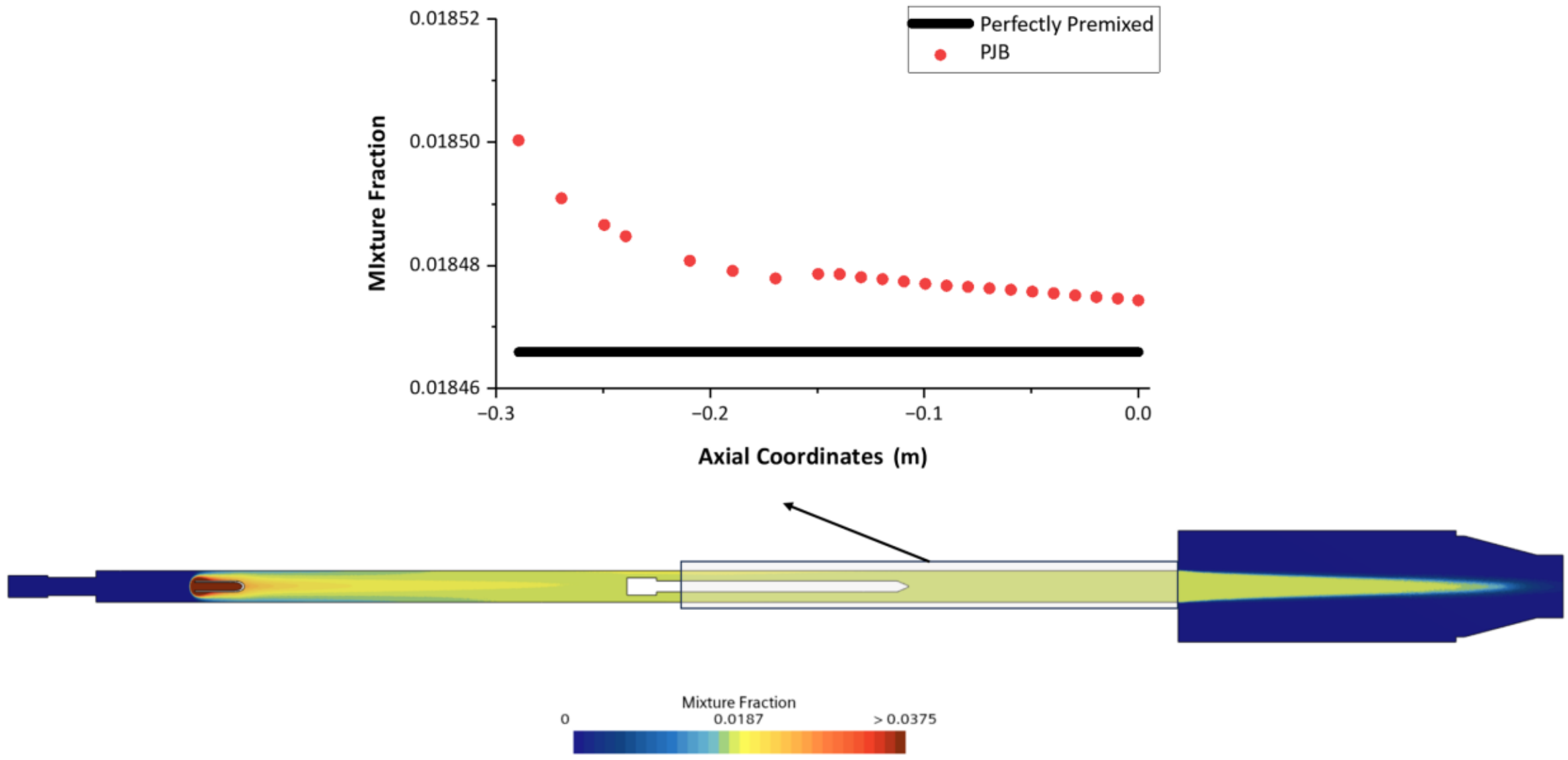

Figure S5 *- Mixture fraction change along the premixing section of the PJB (flow from left to right). The point at the 0 axial coordinate marks where the premixing tube connects to the combustion chamber.*

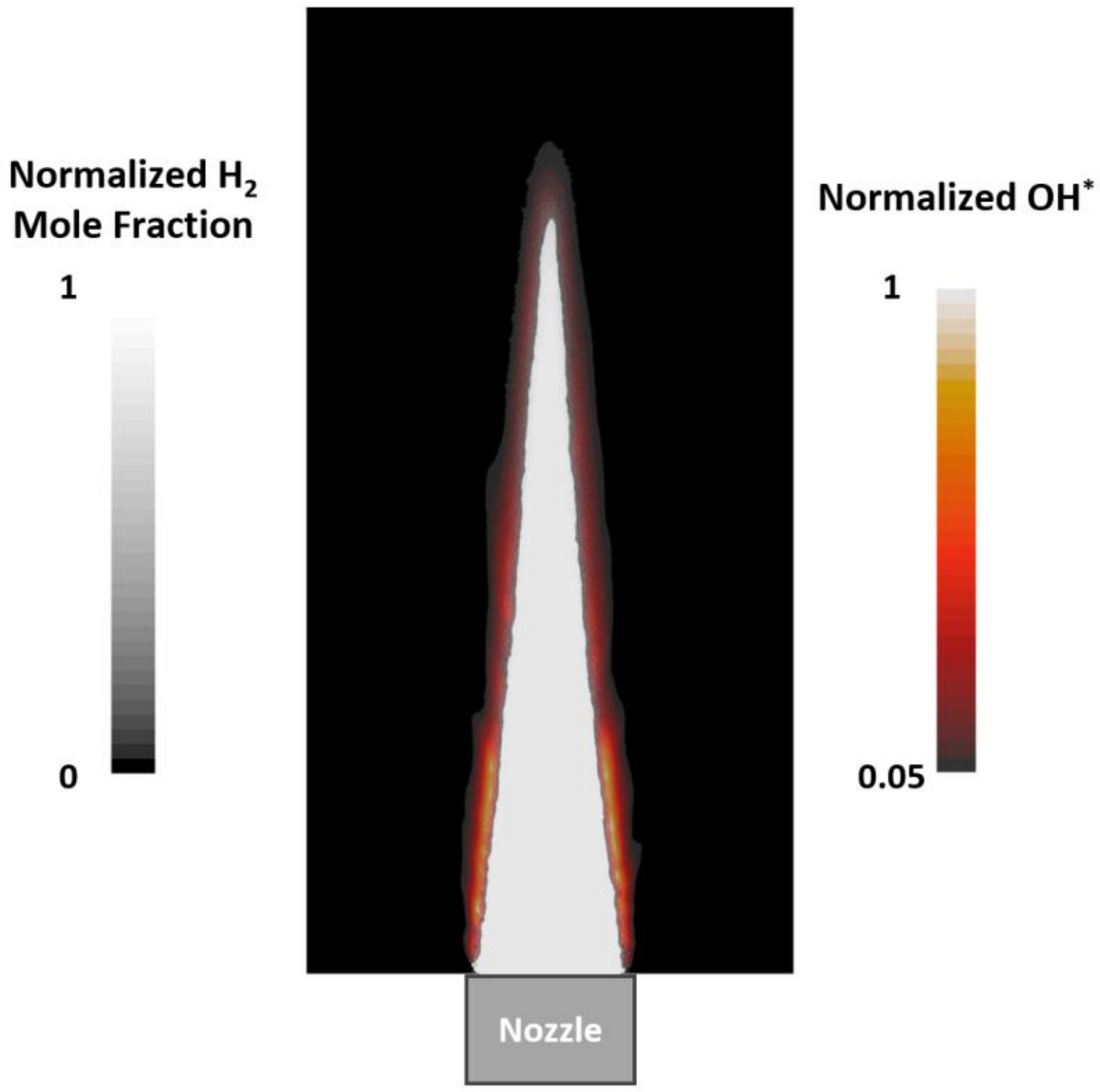

Figure S6 *- Normalized* H_2 *distribution with overlayed* OH^* *distribution for the smooth-wall baseline case at 25 kW,* $\varphi = 0.7$*.* *OH* values below 0.05 are clipped to reduce low-level noise and improve visualization of the flame envelope.*
